# Clustering of adolescents’ health behaviors before and during the COVID-19 pandemic: examining transitions and the role of demographics and parental health behaviors

**DOI:** 10.1186/s12889-025-25368-3

**Published:** 2025-12-06

**Authors:** Nina van den Broek, Linnea Cederlund, Emma Koenders, Renske van der Cruijsen, Junilla K. Larsen, Roy Otten, Jacqueline M. Vink

**Affiliations:** Behavioural Science Institute, Radboud University, P.O. Box 9104, Nijmegen, 6500 HE The Netherlands

**Keywords:** COVID-19 pandemic, Health behaviors, Person-centered analyses, Adolescence, Parents

## Abstract

**Background:**

The COVID-19 pandemic and its measures have profoundly affected adolescents’ lives, including their health behaviors. As a host of research indicates that adolescent health behaviors cluster together and that adolescents can respond differently to the pandemic, we examined adolescents’ changes in clusters of health behaviors and its correlates during different phases of the pandemic.

**Methods:**

A total of 710 Dutch adolescents (53% female; at pre-pandemic: *M*_age_ = 14.37; *SD*_age_ = 0.65; age range = 12.72 to 17.06 years) completed questionnaires on health behaviors (i.e., nicotine use, alcohol use, unhealthy food intake, and physical inactivity) before the pandemic (spring 2019) and during different phases of the pandemic (lockdown 1 (spring 2020), reopening 1 (fall 2020), lockdown 2 (spring 2021), reopening 2 (fall 2021)). We used a person-centered technique to explore how health behaviors clustered in latent classes before and during different phases of the pandemic, and whether adolescents transitioned to different classes during different phases. Adolescent demographics (i.e., age, educational type, and sex) and parental health behaviors were tested as covariates of class membership and transitions.

**Results:**

The Latent Transition Analysis (LTA) identified three classes before the pandemic: one class characterized by health-risk substance use and food intake, one class characterized by health-protective versions of these behaviors, and a medium health-risk class. During the pandemic phases, two classes were identified: most adolescents in the health-risk or health-protective class transitioned to a class with similar health-risk and health-protective behaviors. The third, medium health-risk, class was split into a health-protective and a health-risk class during the pandemic, with most adolescents transitioning to the health-risk class. Age, educational type, sex, and parental food intake behavior were related to latent class membership at some, but not all, time points. Some parental health behaviors, but not adolescent demographic factors, were related to the transition from the medium health-risk to the health-risk class.

**Conclusions:**

Adolescents at-risk for unhealthy behaviors may remain so during the pandemic. Adolescent demographics and parental (food intake) behaviors are relevant to consider as forms of screening adolescents who may be more at risk to show health-risk behaviors. Larger studies are needed to corroborate these findings.

**Supplementary Information:**

The online version contains supplementary material available at 10.1186/s12889-025-25368-3.

The COVID-19 pandemic and its measures have profoundly affected adolescents’ lives [1], as their schools were closed and they were largely confined to their homes [2]. Additionally, sports activities with peers were suspended, school canteens, restaurants, and cafes were closed, and events (e.g., parties and festivals) were cancelled. Parents spent more time at home during the pandemic [2], during a period when adolescents form health habits that shape their current and future health and well-being [3, 4]. Studies suggest that the COVID-19 pandemic and its measures affected adolescents’ health behaviors, although the direction of effects is mixed, with studies reporting both positive and negative effects [5–9]. Hence, it is critical to further study these COVID-induced changes in adolescents’ health behaviors and for whom they occurred. In this paper, we provide insights into changes in adolescents’ health behaviors and their correlates during different phases of the COVID-19 pandemic, offering two key contributions to the field.

First, previous studies addressing the impact of the pandemic (and its associated measures) have mostly examined different health behaviors in isolation. Specifically, research has assessed the pandemic’s effects on nicotine use, alcohol use, unhealthy food intake, and physical inactivity separately [6, 7, 10, 11],van den [12, 13]. These are relevant behaviors, as they represent key modifiable risk factors for non-communicable diseases, such as cardiovascular diseases, diabetes, and several types of cancer [14]. However, a substantial body of research shows that these behaviors do not occur in isolation, but often co-occur or cluster [15–20]. For instance, a systematic review found that people who use nicotine are also more likely to use alcohol, while unhealthy food intake and physical inactivity frequently co-occur [16]. Since clustered health-risk behaviors are more harmful than single behaviors [21, 22], it is critical to study how these patterns may have changed during the pandemic. Therefore, in the present study, we examined the impact of the COVID-19 pandemic on adolescents’ nicotine use, alcohol use, unhealthy food intake, and physical inactivity simultaneously using a clustering approach.

Second, previous research addressing the impact of the COVID-19 pandemic has generally taken a variable-centered approach, assessing whether the pandemic was associated with a general decrease or increase in specific health-risk behaviors [6, 7, 10–13]. This approach assumes that the same changes in a variable apply to all adolescents. However, it can be theorized that adolescents respond differently to the COVID-19 pandemic and its associated measures. Illustratively, for some adolescents, home confinement, stress, and boredom (induced by the COVID-19 pandemic and its measures) may have caused an increase in unhealthy behaviors [23, 24]. For others, spending less time outside the home might have reduced the opportunities and temptations to engage in unhealthy behaviors, resulting in reduced health-risk behaviors [12]. This may (partly) explain why the results of studies using a variable-centered approach to assess the pandemic’s effects on individual health have been rather mixed [5–9]. To acknowledge heterogeneity, we used a person-centered approach that allows the identification of different latent subgroups of adolescents showing different (changes in) health behavior patterns, rather than a variable-centered approach where average changes in health behaviors are assumed to apply to all adolescents [25–27].

So far, two studies have addressed the impact of the COVID-19 pandemic on multiple health behaviors simultaneously using a person-centered approach in (young) adults. In one study involving adults ([28], pre-print at the time of writing), five distinct subgroups were identified, each comprising individuals with similar patterns of change based on five health behaviors (i.e., frequency of alcohol consumption, binge drinking, smoking, body mass index, and sleep) across three timepoints during the first nine months of the pandemic. Additionally, relevant risk and protective predictors of subgroup membership, such as demographics and adverse life events, were identified. In another study of university students and staff [18], four clusters emerged -one lower health-risk, and three higher health-risk- based on six health behaviors (i.e., past-week alcohol, binge-drinking, tobacco, marijuana, benzodiazepine use, and unhealthy food consumption) over a pre-pandemic and six-month COVID-period. Furthermore, changes in cluster membership were uniquely linked to mental health indicators [18]. However, to our knowledge, no studies have applied this approach in adolescent samples, and the two existing studies covered only a short part of the pandemic (i.e., the first six and nine months), limiting insights into longer-term effects on adolescents. Therefore, we examined several adolescent health behaviors across four phases of the COVID-19 pandemic spanning a longer time frame of 1.5 years.

In addition to better understanding the clustering of adolescents’ health behaviors over time, it is important to gain insight into which adolescents are most likely to belong to these clusters, and which are most likely to change cluster membership across different phases of the COVID-19 pandemic. Several demographic factors (e.g., age, educational type, and sex) may help explain differences in adolescents’ health behaviors and changes therein. Regarding age, before the pandemic, the prevalence of health risk behaviors generally increased with age [29]. There are also indications that during the pandemic, older people were more likely to engage in health-risk behaviors than younger people [28]. With respect to educational type, students in pre-vocational education were more likely to engage in health-risk behavior than students in pre-university education [30]. Some researchers even refer to an “education gap” in health behavior [31]. Regarding sex differences, studies indicate that while males are more physically active (van den [32]), females seem more likely to follow a healthier diet [33]. Hence, we exploratorily examined the association between these three demographic factors (i.e., age, educational type, and sex) and adolescents’ (changes in) clusters of health behaviors, to better understand which adolescents are most at risk engaging in or transitioning to health-risk behaviors during various pandemic phases.

Beyond demographic factors, parental factors may also be associated with (changes in) health behaviors. During the pandemic in general, and especially during lockdowns, adolescents and their parents were required to follow education or work from home for extended periods [2]. As a result, adolescents and their parents spent more time together at home [1, 34]. It is possible that parental health behaviors exerted greater influence during this time, as research indicates that modeling increases with more exposure [35]. This is in line with the social learning theory [36], which posits that people can adopt the behavior of others through observation. Alternatively, since adolescence is a developmental phase in which gaining independence is key, increased time spent with parents could put strain on the parent–child relationship and interactions may be under pressure [37]. Potential conflicts and a lower parent–child relationship quality may result in diminished parent–child resemblance in health behaviors [38]. Hence, we exploratorily investigated the association between parental health behaviors and adolescents’ (changes in) clusters of health behaviors during the pandemic.

## The current study

In this study, we first aimed to provide insights into changes in adolescents’ health behaviors during the COVID-19 pandemic. By applying a person-centered technique latent transition analysis (LTA), we could explore how multiple health behaviors (i.e., nicotine use, alcohol use, unhealthy food intake, and physical inactivity) clustered in latent classes before and during different phases of the pandemic, and whether adolescents transitioned between these classes across the various phases of the pandemic. In this way, we addressed the research question: (RQ1) How did latent class membership based on adolescents’ health behaviors change over the course of the COVID-19 pandemic compared to before the pandemic? The second aim was to assess the correlates of adolescents’ health behaviors during the COVID-19 pandemic. Hence, we addressed the research questions: To what extent was latent class membership based on adolescents’ health behaviors associated (RQ2) with demographics and (RQ3) with parental health behaviors? Given the exploratory nature of this study, we did not formulate specific hypotheses.

## Method

The research questions, procedures, and analytic plan were pre-registered on the Open Science Framework (OSF; https://osf.io/bvfxr). The deviations from our pre-registration can be found in Supplemental Text A.

### Procedure

Participants in this study were part of the “G(F)OOD together” research project, a 6-wave longitudinal study on Dutch adolescents’ and their parents’ health behavior ([12, 35], https://osf.io/bysgq/). Wave 1 to 3 were conducted before the COVID-19 pandemic (fall 2017, spring 2018, and spring 2019), and Wave 4 to Wave 6 were conducted during the pandemic (i.e., fall 2020, spring 2021, and fall 2021; see Supplemental Figure A). Furthermore, at Wave 4, adolescents and their parents also retrospectively reported on their behaviors during the first Dutch lockdown (Wave 4 retro, Spring 2020), providing an additional timepoint. Adolescents and their parents were recruited through seven Dutch secondary schools in the Netherlands. In this study, we included data of Wave 3 (pre-pandemic) and onwards (during the COVID-19 pandemic).

This longitudinal survey study was conducted in accordance with the Declaration of Helsinki, and was approved by the Ethics Committee Social Sciences of Radboud University, Nijmegen, The Netherlands (ECSW-2017–0805-516; ECSW-2020–122; ECSW-2020–182; ECSW-2021–043). Parents provided active consent for their child’s and their own participation, after which adolescents additionally provided consent to participate. In each wave, adolescents and their parents completed an online survey via Qualtrics Survey Software. At Wave 3, adolescents completed the survey at school during one classroom session (approximately 50 min) with the researchers present. At Waves 4 to 6, due to the COVID-19 restrictions, adolescents completed the survey independently on their own devices, as researchers could not visit schools. Parents completed all questionnaires online. Participants received a small gift, gift voucher, or weekend getaway to thank them for their participation. More details on the procedures are available in our pre-registration.

### Participants

We included data from both adolescents and parents who participated in any of the Waves 3–6 of the “G(F)OOD together” project. Data from 12 non-biological parents and 2 parents with missing relationship information were excluded. In some cases, both biological parents participated. To pair adolescents with a single parent and maximize inclusion of fathers, 66 mothers were excluded in cases in which also fathers participated. The final analytical sample consisted of *N* = 710 adolescents (53% female), *N* = 328 biological mothers, and *N* = 121 biological fathers.

Due to attrition, the number of participants varied across waves (Supplemental Table B). All adolescents (Wave 3: *M*_age_ = 14.37; *SD*_age_ = 0.65; age range = 12.72 to 17.06 years) included in the study attended secondary education. Of these, 274 were enrolled in pre-vocational training (i.e., “vmbo”; lower type), and 436 attended higher general secondary education or pre-university secondary education (i.e., “havo” or “vwo”; higher type). The mean age of biological mothers at Wave 3 was 45.90 years (*SD*_age_ = 4.20, age range = 31.20 to 57.10 years). Of these mothers, 38.6% completed a lower type of education (i.e., elementary school, prevocational secondary education (“vmbo”), or secondary vocational education (“mbo”)) and 61.4% completed a higher type of education (i.e., higher general secondary education (“havo”), pre-university secondary education (“vwo”), applied university education (“hbo”), or university education (“wo”)). The mean age of biological fathers at Wave 3 was 49.00 years (*SD*_age_ = 4.20, age range = 39.20 to 62.80 years) and 64.0% completed a higher educational type. Attrition analysis comparing adolescents who dropped out after the pre-pandemic wave (*N* = 373) to those who participated in at least one pandemic wave (*N* = 301) showed that attrition is more prevalent in older adolescents and those who use alcohol infrequently (compared to younger adolescents and alcohol abstainers). No differences were found on the other health behaviors and demographics, suggesting rather limited evidence of systematic attrition (see Supplemental Table C).

### Measures

To assess the four health behaviors, we used eight indicators for both adolescents and parents: nicotine use (indicators: cigarette use and e-cigarette use), alcohol use, food intake (indicators: sugar sweetened beverages (SSBs) intake, sweet snack intake, savory snack intake, fruit and vegetables intake), and physical activity. Categorical variables were created to use the indicators in the analyses, in order to appropriately reflect the ordinal structure of the substance use response scales and to address the non-normal distributions observed in the food intake responses. Table 1 and Supplemental Text B provide information on the measures, which were based on previous work but adapted for use in the longitudinal study, and recoding procedures used for these indicators.

**Table 1:** Health Behaviors Indicators Used for the Latent Class and Transition Analyses

Health behavior indicator	Original item(s)	Original Response Option (ORO)	Categories used in the LTA
Cigarette use	Which statement about smoking applies most to you?	1. I have never smoked, not even a puff 2. I have taken a puff of a cigarette a few times 3. I used to smoke (regularly), but I have quit smoking now 4. I smoke once a week or less 5. I smoke several times a week 6. I smoke daily	1. Non-Smokers = ORO 1 and 3 2. Experimental Smokers = ORO 2 3. Infrequent Current Smokers = ORO 4 4. Frequent Current Smokers = ORO 5 and 6
E-cigarette use	How often do you use e-cigarettes?	Wave 3: 0, 1, 2, 3, 4, 5, 6, 7, 8, 9, 10, 11 to 19, 20 to 39, 40 + Other waves: Never, Sometimes, Regularly	1. Non-Users: ORO 0 (Wave 3) or Never (other waves) 2. Users: ORO > 0 (Wave 3) or Sometimes or Regularly (other waves)
Alcohol use	Which statement about alcohol applies most to you?	1: I have never drunk alcohol, not even a sip 2: I have had a sip of alcohol a few times 3: I used to drink alcohol (regularly), but I don't drink anymore 4: I drink alcohol once a week or less 5: I drink alcohol several times a week 6: I drink alcohol daily	1. Non-Drinkers: ORO 1 and 3 2. Experimental Drinkers = ORO 2 3. Infrequent Current Drinkers = ORO 4 4. Frequent Current Drinkers = ORO 5 and 6
SSBs intake	During a regular week, how often do you drink soft drinks? In Waves 3, 4 retro, and 4, separately for a) taken or received from home; b) bought at school; c) bought somewhere else; and d) received somewhere else (*four contexts*)	0 days per week to 7 days per week (8 options)	For waves 3, 4 retro, and 4: A maximum number of days per week was determined, ranging from 0 (Category 1) to 7 days (Category 8) by selecting the highest value reported across the four contexts
Sweet snack intake	During a regular week, how often do you eat: 1. cake, 2. candy bars, 3. chocolate (*three questions*)? In Waves 3, 4 retro, and 4, separately for a) taken or received from home; b) bought at school; c) bought somewhere else; and d) received somewhere else (*four contexts*)	0 days per week to 7 days per week (8 options)	A maximum number of days per week was determined, ranging from 0 (Category 1) to 7 days (Category 8) by selecting the highest value reported across the four contexts (for waves 3, 4 retro, and 4) and the three types of sweet snacks
Savory snack intake	During a regular week, how often do you eat warm, fried snacks? In Waves 3, 4 retro, and 4, separately for a) taken or received from home; b) bought at school; c) bought somewhere else; and d) received somewhere else (*four contexts*)	0 days per week to 7 days per week (8 options)	For waves 3, 4 retro, and 4: A maximum number of days per week was determined, ranging from 0 (Category 1) to 7 days (Category 8) by selecting the highest value reported (across the four contexts)
Fruit and vegetables intake	During a regular week, how often do you eat: 1. fruit, 2. raw vegetables, 3. warm vegetables (*three questions*)? In Waves 3, 4 retro, and 4, separately for a) taken or received from home; b) bought at school; c) bought somewhere else; and d) received somewhere else (*four contexts*)	0 days per week to 7 days per week (8 options)	A maximum number of days per week was determined, ranging from 0 (Category 1) to 7 days (Category 8) by selecting the highest value reported across the four contexts (for waves 3, 4 retro, and 4) and the three types of fruit and vegetables
Physical activity	Participants were asked to indicate the frequency and amount for moderate activity and vigorous activity	Frequency of activity: 0 days per week to 7 days per week (8 options) In case Frequency ≠ 0: Amount of physical activity in free form (Wave 3) or Likert scale (other waves)	1. Insufficiently active: less than 420 min per week (children) or less than 150 min per week (parents) on moderate/vigorous physical activity 2. Sufficiently active: at least 420 min per week (children) or at least 150 min per week (parents) on moderate/vigorous physical activity

Further details on measurement and recoding can be found in Supplemental Text B

*SSBs *Sugar-sweetened beverages,* Retro *Retrospective

The framing of the indicator questions varied across waves. For e-cigarette use, covariates, and Wave 3, participants reported behaviors without reference to any specific COVID-19 period. Wave 4 retrospective (retro) and Wave 4 were part of the same questionnaire, in which adolescents and parents were asked to report on behavior “during the first lockdown” (Wave 4 retro) and “since the start of school year” (Wave 4). For Wave 5, participants reported behaviors “since the second lockdown”, and for Wave 6, behaviors over “the past six months”. For clarity, we refer to W3 as pre-pandemic, W4 retro as lockdown 1, W4 as reopening 1, W5 as lockdown 2, and W6 as reopening 2.

### Data-analysis

Latent Transition Analysis (LTA) was conducted using the first four steps of the five-step approach [26, 39, 40]. The LTA analyses steps were performed in Mplus version 8 [41]) using the Robust Maximum Likelihood estimator (MLR) and Full Information Maximum Likelihood (FIML) to deal with potentially non-normality and missingness. R [42] was used for preparations and descriptives and a part of Step 4. Our R scripts, Mplus input files, and Mplus output files can be found on OSF (https://osf.io/8yznm/files). Our analytic datasets can be found on the Radboud Data Repository (10.34973/eekd-9v39).

#### Step 0: descriptive statistics

Frequency tables for the main measures for each wave were generated using R. In addition, we computed both Pearson’s and Spearman’s correlation coefficients between the different health behavior indicators and demographic covariates in R, to determine which demographic covariates should be included in Step 4.

#### Step 1: study measurement model alternatives for each time point

In Step 1, to determine the number of classes that described the heterogeneity in health behaviors in the sample most optimally, we conducted separate Latent Class Analysis (LCA) for each wave. To identify the optimal number of classes, we used the Akaike Information Criterion (AIC), sample-size adjusted Bayesian Information Criterion (aBIC), the Vuong-Lo-Mendell-Rubin Likelihood Ratio Test (VLMR-LRT), and the Bootstrapped Likelihood Ratio Test (BLRT) [43, 44] and classification diagnostics for models with the best fit [45], see Supplemental Text C for more details. The meaning of the latent classes was interpreted using latent class-specific item-response probabilities, which represent the likelihood of adolescents engaging in each health behavior within a class.

#### Step 2: explore transitions based on cross-sectional results and measurement invariance

In Step 2, adolescents were assigned to the latent class with the highest posterior probability, at each time point. To gain a preliminary understanding of class transitions, we cross-tabulated latent class memberships across time points. In this step, we also conducted formal tests of measurement invariance, see Supplemental Text C for more details.

#### Step 3: explore specifications of the LTA without covariates

In Step 3, we conducted the LTA model and obtained transition probabilities between the latent classes. Structural, autoregressive paths from the latent class variables at *t* were added to *t* + 1, which enabled the estimation of transition probabilities. The transition probabilities represent the likelihood of an individual to move from one latent class to another at each time point. We used the three-step approach introduced by Vermunt [46] to specify the LTA, of which further details can be found in Supplemental Text C.

#### Step 4: include covariates in the LTA model

In Step 4, we included the covariates in the LTA model as predictors. In this step, we included time-invariant covariates that did not have missing values, because Mplus with FIML cannot handle missing data in the covariates. Thus, the demographics age, educational type, and sex (which contained no missings) were included at this step. We investigated both the effect of the covariates on class membership (i.e., main effect) and transition probabilities (i.e., interaction effect). The eight parental health behavior indicators were associated with adolescents health behavior classes using multinomial logistic regressions (in case of 3 or more classes) or logistic regressions (in case of 2 classes) in R, as these variables contained missings. As these models estimate a single outcome, we ran separate (i.e., univariate) models with class membership per COVID-19 timepoint as the outcome. In the analyses in R, FIML was not available to handle missingness using the multinom() and glm() functions and hence, only complete cases were used.

## Results

### Step 0: descriptive statistics

The percentage of adolescents in each category of the eight health behavior indicators are presented in Supplemental Table D. Percentages indicate that cigarette and alcohol use become more prevalent over time, while e-cigarette use remains stable (i.e., low). Consumption of sweet and savory snacks appears to decrease slightly over time. Physical activity shows marked variation, with fewer adolescents being sufficiently active during periods of lockdowns compared to non-lockdowns. Supplemental Table E shows the bivariate correlations between the health behavior indicators and demographics. Notably, particularly at the first timepoints, age, educational type, and sex were related to specific health behaviors. Given these relevant associations, we included all three factors as covariates in Step 4 of the LTA.

### Step 1: study measurement model alternatives for each time point

Results of the LCA (see Table 2) suggest that the 3- and 4-class model were the most plausible at the pre-pandemic timepoint, given the combination of the lowest values of the fit indices and significance of the BLRT. In the end, the 3-class solution for the pre-pandemic period was selected, given that the aBIC showed no improvement in the 4-class model, the 4-class model had one very small class (below 5%), and we had pre-registered to choose to most parsimonious model in such cases. At the timepoints during the pandemic, the 2- and 3-class solutions were most plausible. For these timepoints, we chose the 2-class solutions given the lower aBIC and/or AIC and for reasons of parsimony. Entropy and average class assignment probability were sufficient.

**Table 2:** Fit Indices for Different Number of Classes Across the Five Timepoints

# classes	Par	LL	AIC	aBIC	VLMR-LRT (*p*)	BLRT (*p*)	Entropy	Percentage smallest class	Average class assignment probability per class								
Pre-pandemic																	
1	36	−5,785.26	11,642.52	11,690.70	n.a	n.a	n.a	n.a	n.a								
2	73	−5,631.93	11,409.87	11,507.55	1.000	.000	.72	23%	.89					.93			
3	110	−5,554.10	11,328.21	11,475.40	.817	.000	.70	8%	.83		.92					.86	
4	147	−5,513.74	11,321.48	11,518.20	.849	.002	.77	4%	.92	.96			.84				.85
Lockdown 1																	
1	36	−2,678.04	5,428.10	5,447.61	n.a	n.a	n.a	n.a	n.a								
2	73	−2,621.53	5,389.07	5,428.65	.265	.000	.71	27%	.87				.93				
3	110	−2,580.35	5,380.71	5,440.36	.766	.004	.74	6%	.86			.94			.89		
4	147	−2,545.79	5,385.58	5,465.29	.792	.076	.80	4%	.98	.91			.89				.85
Reopening 1																	
1	36	−2,650.52	5,373.03	5,392.55	n.a	n.a	n.a	n.a	n.a								
2	73	−2,581.12	5,308.25	5,347.83	.002	.000	.73	28%	.86				.94				
3	110	−2,545.73	5,311.46	5,371.11	.784	.048	.76	19%	.93			.91			.87		
4	147	−2,516.56	5,327.12	5,406.83	.805	.326	.80	12%	.92	.85			.94				.85
Lockdown 2																	
1	36	−1,230.24	2,532.48	2,523.97	n.a	n.a	n.a	n.a	n.a								
2	73	−1,189.67	2,525.35	2,508.09	.219	.001	.82	26%	.92				.95				
3	110	−1,161.37	2,542.74	2,516.73	1.000	.204	.92	10%	.95			.97			.96		
4	Did not converge																
Reopening 2																	
1	36	−1,163.95	2,399.90	2,388.72	n.a	n.a	n.a	n.a	n.a								
2	73	−1,125.34	2,396.68	2,374.01	.704	.002	.77	50%	.95				.94				
3	110	−1,096.01	2,412.01	2,377.85	.779	.170	.87	20%	.95			.99			.88		
4	Did not converge																

Selected number of classes are highlighted in grey

*Par* Number of free parameters, *LL* Loglikelihood value, *AIC* Akaike Information Criterion, *aBIC* Sample-Size Adjusted Bayesian Information Criterion, *VLMR-LRT* Vuong-Lo-Mendell-Rubin Likelihood Ratio Test, *BLRT* Bootstrapped Likelihood Ratio Test, *n.a.* not applicable

**Fig. 1:**
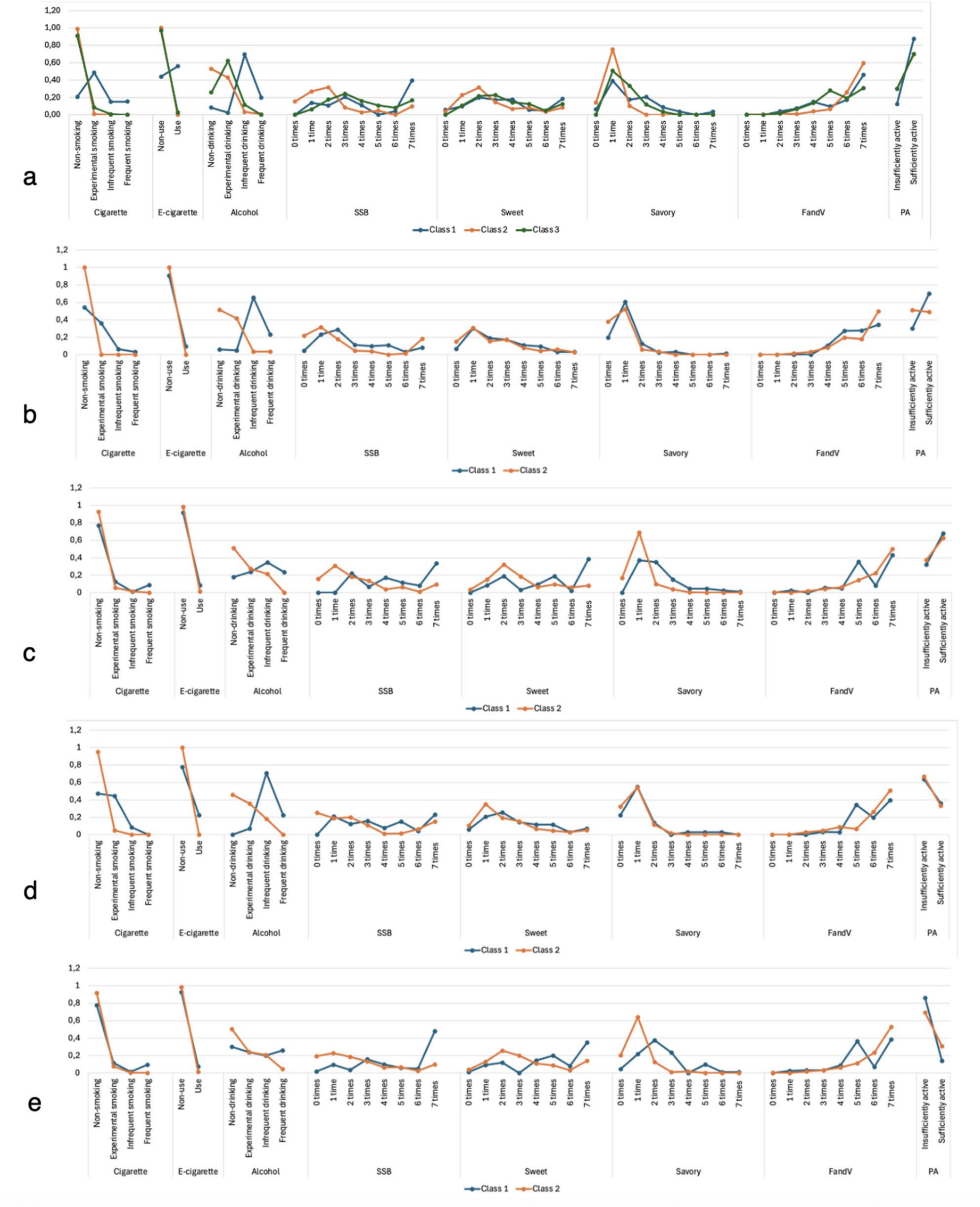
Visualizations of the Item-Response Probabilities (see Supplemental Excel File) Across the Identified Classes at (**a**) Pre-Pandemic, (**b**) Lockdown 1, (**c**) Reopening 1, (**d**) Lockdown 2, and (**e**) Reopening 2. SSB = Sugar-Sweetened Beverages; FandV = Fruit and Vegetables; PA = Physical Activity

We named the classes using the item-response probabilities (as visualized in the Supplemental Excel File and Figs. 1a-e). At the *pre-pandemic* timepoint (Fig. 1a and Supplemental Excel Figure A), Class 1 showed a relatively high level of substance use and unhealthy food intake, but also high physical activity (“Health-risk substance use and food, health-protective physical activity” class). In contrast, Class 2 showed little to no substance use, relatively low unhealthy food intake, and -although less than Class 1- still relatively high physical activity (“Health-protective” class). Class 3 showed a pattern between Class 1 and 2, with some substance use, some unhealthy food intake, and -though less than in Class 1-, still a sufficient level of physical activity (“Medium health-risk” class).

At *lockdown 1* (Fig. 1b and Supplemental Excel Figure B), physical activity was found to be substantially lower in both classes, compared to the pre-pandemic period. Adolescents in Class 1 showed more substance use and unhealthy food intake and physical inactivity compared to adolescents in Class 2 (“Health-risk” class). Class 2 was characterized by low use of substances and low unhealthy food intake, but adolescents were physically inactive, though less so than those in Class 1 (“Health-protective substance use and food, health-risk physical activity” class).

*Reopening 1* (Fig. 1c and Supplemental Excel Figure C) revealed similar patterns to lockdown 1, but with higher levels of physical activity in both classes (although still below pre-pandemic levels). Class 1 showed more physical activity than Class 2, and physical activity overall was relatively high compared to lockdown periods. Therefore, we named Class 1 as the “Health-risk substance use and food, health-protective physical activity” class, and Class 2 as the “Health-protective” class.

At *lockdown 2* (Fig. 1d and Supplemental Excel Figure D), consistent with lockdown 1, physical activity was again much lower in both classes, compared to pre-pandemic. Class 1 showed considerable substance use and unhealthier eating patterns, although this was less pronounced than at previous timepoints (“Health-risk” class). Class 2 showed relatively little substance use and unhealthy food intake (“Health-protective substance use and food, health-risk physical activity”).

At *reopening 2* (Fig. 1e and Supplemental Excel Figure E), physical activity was higher than during lockdown 2, but on average still lower than at pre-pandemic. Class 1 showed substantial substance use, unhealthier food intake (though less pronounced than at the previous timepoints), but more physical activity than Class 2 (“Health-risk substance use and food, health-protective physical activity” class). Class 2 showed little substance use and unhealthy food intake, but remained relatively physically inactive (“Health-protective substance use and food, health-risk physical activity” class).

### Step 2: explore transitions based on cross-sectional results and measurement invariance

In Step 2, adolescents were assigned to their most likely latent class using modal class assignment. Cross-tabulations of class membership over time are presented in Tables 3, 4, 5, and 6, showing that adolescents in the higher health-risk classes (in terms of substance use and food intake) were more likely to drop-out over time. To evaluate the appropriateness of testing for measurement invariance, we examined the item-response probabilities across the last four last waves (where the number of classes remained the same) to assess similarity of class structure over time (see Supplemental Excel Figures F to M). Based on the inspection, we concluded that the classes were not similar enough to be considered the same across time. Therefore, we decided to perform the LTA with the unconstrained (fully variant) model.

**Table 3:** Cross-Tabulation of Cross-Sectional Transitions from Pre-Pandemic to Lockdown 1 (*N* = 674)

		Lockdown 1						
		Class 1^d^		Class 2^e^		Missing		
		*n*	%	*n*	%	*n*	%	Sum
Pre-pandemic	Class 1^a^	8	16.3	4	8.2	37	75.5	49
	Class 2^b^	16	4.9	137	41.8	175	53.4	328
	Class 3^c^	49	16.5	72	24.2	176	59.3	297

^a^Health-risk substance use and food, health-protective physical activity class, ^b^Health-protective class, ^c^Medium health-risk class, ^d^Health-risk class, ^e^Health-protective substance use and food, health-risk physical activity class

**Table 4:** Cross-Tabulation of Cross-Sectional Transitions from Lockdown 1 to Reopening 1 (*N *= 303)

		Reopening 1						
		Class 1^c^		Class 2^d^		Missing		
		*n*	%	*n*	%	*n*	%	Sum
Lockdown 1	Class 1^a^	53	67.9	25	32.1	0	0	78
	Class 2^b^	32	14.2	193	85.8	0	0	225
	Sum	73		213		0	0	303

^a^Health-risk class, ^b^Health-protective substance use and food, health-risk physical activity class, ^c^Health-risk substance use and food, health-protective physical activity class, ^d^Health-protective substance class

**Table 5:** Cross-Tabulation of Cross-Sectional Transitions from Reopening 1 to Lockdown 2 (*N* = 303)

		Lockdown 2						
		Class 1^c^		Class 2^d^		Missing		
		*n*	%	*n*	%	*n*	%	Sum
Reopening 1	Class 1^a^	10	11.8	9	10.6	66	77.6	85
	Class 2^b^	14	6.4	71	32.6	133	61.0	218
	Sum	24		80		199		303

^a^Health-risk substance use and food, health-protective physical activity class, ^b^Health-protective class, ^c^Health-risk class, ^d^Health-protective substance use and food, health-risk physical activity class

**Table 6:** Cross-Tabulation of Cross-Sectional Transitions from Lockdown 2 to Reopening 2 (*N* = 138)

		Reopening 2						
		Class 1^c^		Class 2^d^		Missing		
		*n*	%	*n*	%	*n*	%	Sum
Lockdown 2	Class 1^a^	23	69.7	0	0	10	30.3	33
	Class 2^b^	28	26.7	61	48.1	16	15.2	105
	Sum	51		61		26		138

^a^Health-risk class, ^b^Health-protective substance use and food, health-risk physical activity class, ^c^Health-risk substance use and food, health-protective physical activity class, ^d^Health-protective substance use and food, health-risk physical activity class

### Step 3: Explore Specifications of the LTA without Covariates

The LTA model was conducted to estimate transition probabilities using the three-step approach proposed by Vermunt [46]. Tables 7, 8, 9, 10 show the transition probabilities reflecting the probability of membership in each latent class at time *t* + 1 given the latent class membership at *t*. The transition probabilities indicated that across the pandemic waves, most adolescents that were in a class with health-protective substance use and food intake transitioned to a health-protective substance use and food intake class in the succeeding wave (probabilities between 0.69 and 0.96). Most adolescents that were in a class with higher health-risk substance use and food intake transitioned to a higher health-risk substance use and food intake class in the succeeding wave as well (probabilities between 0.73 and 1.00). Similarly, transition probabilities from pre-pandemic to lockdown 1 indicated a similar pattern (probabilities of 0.98 and 0.80 for transition to and from health-protective substance use and food intake, and from and to health-risk substance use and food intake, respectively). Furthermore, 58% of adolescents that were in the medium health risk class at pre-pandemic, transitioned to the health-risk substance use and food intake class at lockdown 1, while the rest (42%) thus transitioned to the health-protective substance use and food class.

**Table 7:** Transition Probabilities from Pre-pandemic to Lockdown 1 (N = 710)

		Lockdown 1	
		Class 1^d^	Class 2^e^
Pre-pandemic	Class 1^a^	0.80	0.20
	Class 2^b^	0.02	0.98
	Class 3^c^	0.58	0.42

^a^Health-risk substance use and food, health-protective physical activity class, ^b^Health-protective substance class, ^c^Medium health-risk class, ^d^Health-risk class, ^e^Health-protective substance use and food, health-risk physical activity class

**Table 8:** Transition Probabilities from Lockdown 1 to Reopening 1 (*N* = 710)

		Reopening 1	
		Class 1^c^	Class 2^d^
Lockdown 1	Class 1^a^	0.85	0.15
	Class 2^b^	0.04	0.96

^a^Health-risk class, ^b^Health-protective substance use and food, health-risk physical activity class, ^c^Health-risk substance use and food, health-protective physical activity class, ^d^Health-protective class

**Table 9:** Transition Probabilities from Reopening 1 to Lockdown 2 (*N* = 710)

		Lockdown 2	
		Class 1^c^	Class 2^d^
Reopening 1	Class 1^a^	0.73	0.27
	Class 2^b^	0.16	0.84

^a^Health-risk substance use and food, health-protective physical activity class, ^b^Health-protective class, ^c^Health-risk class, ^d^Health-protective substance use and food, health-risk physical activity class

**Table 10:** Transition Probabilities from Lockdown 2 to Reopening 2 (N = 710)

		Reopening 2	
		Class 1^c^	Class 2^d^
Lockdown 2	Class 1^a^	1.00	0.00
	Class 2^b^	0.31	0.69

^a^Health-risk class, ^b^Health-protective substance use and food, health-risk physical activity class, ^c^Health-risk substance use and food, health-protective physical activity class, ^d^Health-protective substance use and food, health-risk physical activity class

### Step 4: include covariates in the LTA model

#### Demographics

Table 11 and Supplemental Table F show the results of the LTA with the covariates age, educational type, and sex. The results indicate that pre-pandemic (with three classes), there was a significant effect of age, educational type, and sex on class membership. First, with each additional year of age, adolescents had 3.60 times higher odds of being in the health-risk substance use and food intake class than the health-protective class. Second, compared to adolescents in higher types of education, those in lower types of education had 3.33 higher odds of being in the medium health-risk class or 6.00 higher odds of being in the health-risk substance use and food intake class than in the health-protective substance use and food intake class. Third, compared to females, males had 1.90 higher odds of being in the medium health-risk class than in the health-protective substance use and food intake class. At reopening 1, there was a significant effect of age on class membership; with each additional year of age, adolescents had 2.60 times higher odds of being in the health-risk substance use and food intake class than in the health-protective class. At the other timepoints, no significant associations were found.

**Table 11:** Odds Ratios of Demographic Covariates Predicting Class Membership of Adolescents Per Wave (*N* = 710)

		Pre-pandemic		Lockdown 1		Reopening 1		Lockdown 2		Reopening 2	
Class	Covariate	*OR*	*p*	*OR*	*p*	*OR*	*p*	*OR*	*p*	*OR*	*p*
Class 1^a^	Age	3.60	< .001	0.58	.200	2.58	.045	1.28	.546	1.31	.437
	Lower educational type	6.00	< .001	1.40	.563	0.70	.758	0.89	.846	1.45	.529
	Male sex	1.96	.063	0.84	.757	3.40	.320	0.93	.904	0.44	.241
Class 3^b^	Age	1.43	.089								
	Lower educational type	3.33	< .001								
	Male sex	1.90	.010								

The classes with health-protective substance use and food intake (Class 2) are not present because they were used as reference class in the logistic regression. ^a^Class with health-risk substance use and food intake. ^b^Medium health-risk class (at pre-pandemic only). For the binary variables educational type and sex, higher educational type and females were used as reference classes, respectively. Supplemental Table F can be consulted for the detailed statistical output (i.e., *b*, *SE*, and 95% Confidence Interval)

#### Parental health behaviors

Univariate multinomial logistic regressions (Table 12 and Supplemental Table G) indicate that pre-pandemic, parental intake of savory snacks and fruit and vegetables was significantly associated with class membership of adolescents. Higher parental intake of savory snacks was associated with their adolescent child having 1.92 higher odds to be in the medium health risk class than in the health-protective substance use and food intake class. Furthermore, higher parental fruit and vegetables intake was associated with adolescents having lower odds (*OR* = 0.70) to be in the medium health-risk class than the health-protective substance use and food intake class. In other words, each additional day parents eat fruit and vegetables weekly was associated with a 30% lower odds of their adolescent children being in the medium health-risk class compared to the health-protective class. No other significant associations between parental health behaviors and pre-pandemic class membership of adolescents were found. Additionally, logistic regressions (Table 12 and Supplemental Tables H to K) indicate that at reopening 1, parental intake of sugar-sweetened beverages was significantly associated with class membership of adolescents. More specifically, each additional day parents consumed sugar-sweetened beverages weekly was associated with their child having 1.21 times higher odds to be in the health-risk substance use and food intake class compared to the health-protective class. No significant parent–child associations were found for the other timepoints.

**Table 12:** Odds Ratios of Parental Health Behaviors Predicting Class Membership of Adolescents Per Wave (Univariate Analyses; *N* = 88 to 378)

	Pre-pandemic				Lockdown 1		Reopening 1		Lockdown 2		Reopening 2	
	Class 1^a^		Class 3^b^		Class 1^a^		Class 1^a^		Class 1^a^		Class 1^a^	
Parental Health Behaviors	*OR*	*p*	*OR*	*p*	*OR*	*p*	*OR*	*p*	*OR*	*p*	*OR*	*p*
Cigarette use^c^												
Experimental	0.73	.529	0.70	.157	1.64	.691	1.04	.974	0.89	.854	-	-
Infrequent current^d^	-	-	-	-	3.27	.406	3.12	.426	-	-	-	-
Frequent current	0.86	.849	0.91	.823	1.64	.444	1.04	.956	0.72	.778	-	-
Alcohol use^e^												
Experimental	0.44	.304	0.62	.292	1.47	.540	1.60	.499	0.30	.333	1.03	.973
Infrequent current	0.48	.221	0.94	.864	0.54	.269	1.36	.594	0.81	.782	1.23	.753
Frequent current	0.55	.351	1.05	.907	0.95	.921	1.33	.628	2.62	.212	3.40	.078
SSBs intake	1.08	.372	1.09	.071	1.11	.175	1.21	.011	0.87	.201	1.01	.912
Sweet snack intake	1.15	.226	1.02	.799	1.15	.149	1.07	.485	0.81	.175	0.83	.120
Savory snack intake	1.01	.982	1.92	.003	1.58	.126	1.14	.664	0.72	.448	0.77	.382
Fruit & veg intake	0.79	.215	0.70	< .001	0.78	.096	0.77	.078	0.72	.077	1.13	.527
Physical activity^f^	0.78	.678	0.89	.682	0.62	.220	1.25	.600	1.65	.288	1.74	.214

The classes with health-protective substance use and food intake (Class 2) are not present because they were used as reference class in the logistic regression. ^a^Class with health-risk substance use and food intake. ^b^Medium health-risk class (at pre-pandemic only). ^c^Reference level for cigarette use was non-smoking. ^d^Estimates for this level were not reliable because very few parents were infrequent current users. ^e^Reference level for alcohol use was non-drinking. All the food intake variables were continuous. ^f^Effect of sufficiently active versus insufficiently active. E-cigarette use is not present since almost no parents indicated use of e-cigarettes. Supplemental Table G to K can be consulted for the detailed statistical output (i.e., *b*, *SE*, and 95% Confidence Interval)

#### Additional exploratory results

As interactions could not be added to the Mplus models to allow the demographic and parental covariates to predict transition probabilities (see deviations from pre-registration, Supplemental Text A), we explored the effect of covariates and parental health behaviors on one specific transition that stood out: The transition from the medium health-risk class at pre-pandemic to the two classes at lockdown 1. This transition was notable since a) it is the transition from pre-pandemic to the pandemic and b) it is the transition in which three classes are reduced to two classes, with adolescents in the medium health-risk class being split up to the two remaining classes.

The results of the demographic covariates (Table 13) indicated no significant effect of age, educational type, or sex on this transition. When the parental health behaviors were used as correlates (Table 14), it was found that the intake of sugar-sweetened beverages by parents was associated with the transition. Specifically, each additional day parents consumed sugar-sweetened beverages weekly pre-pandemic was associated with their adolescent child having 1.36 times higher odds to transition to the health-risk class at lockdown 1. Alcohol use by parents was also associated with this specific transition; Adolescents whose parents consumed alcohol (versus those whose parents did not) had lower odds to transition to the health-risk class (*OR* = 0.10).

**Table 13:** Logistic Regressions of Demographic Covariates Predicting Transition from Class 3 Pre-Pandemic (Medium Health-Risk Class) to Class 1 Lockdown 1 (Health-Risk Class) of Adolescents (N = 121)

	*b*	*SE*	*p*	*OR*	Confidence Interval	
					2.5%	97.5%
Age^a^	< 0.01	0.35	.995	1.00	0.50	2.01
Educational type^b^	0.27	0.38	.478	1.31	0.62	2.75
Sex^c^	−0.17	0.37	.644	0.84	0.40	1.75

^a^Age at the date of participation in Wave 3. ^b^0 = Low educational type; 1 = High educational type. ^c^0 = male; 1 = female. Nagelkerke* R*^*2*^ = .008

**Table 14:** Logistic Regressions of Parental Health Behaviors Predicting Transition from Class 3 Pre-Pandemic (Medium Health-Risk Class) to Class 1 Lockdown 1 (Health-Risk Class) of Adolescents (N = 70 to 77)

	*b*	*SE*	*p*^e^	Nagelkerke* R*^*2*^	*OR*	Confidence Interval^e^	
						2.5%	97.5%
Cigarette use^a^				0.06			
Experimental	0.15	0.54	.781		1.16	0.39	3.38
Infrequent current	-	-	-		-	-	-
Frequent current	1.86	1.15	.108		6.4	0.87	130.06
E-cigarette use^b^	0.33	1.43	.819	< 0.01	1.39	0.05	35.99
Alcohol use^c^				0.11			
Experimental	−2.49	1.29	.054		0.08	0.01	0.80
Infrequent current	−2.30	1.29	.041		0.10	0.01	0.66
Frequent current	−2.20	1.17	.061		0.11	0.01	0.82
SSBs intake (continuous)	0.29	0.11	.007	0.13	1.36	1.09	1.67
Sweet snack intake (continuous)	0.26	0.16	.108	0.05	1.30	0.95	1.82
Savory snack intake (continuous)	−0.08	0.42	.859	< 0.01	0.93	0.39	2.14
Fruit & veg intake (continuous)	0.29	0.23	.208	0.03	1.34	0.86	2.15
Physical activity^d^	0.02	0.56	.975	0.56	1.02	0.35	3.15

^a^Reference level for cigarette use was non-smoking. ^b^Reference level for e-cigarette use was non-use. ^c^Reference level for alcohol use was non-drinking. ^d^Effect of sufficiently active versus insufficiently active. ^e^In some cases, the statistical significance conclusions of the *p*-values and confidence intervals do not match up, as the confidence intervals are profile-based, and the p-values are Wald-test-based

## Discussion

This exploratory study aimed to increase our understanding of changes in adolescents’ health behaviors and their correlates during the COVID-19 pandemic using a person-centered approach. More specifically, we explored how several health behaviors (i.e., nicotine use, alcohol use, unhealthy food intake, and physical inactivity) clustered into latent classes before and during different phases of the pandemic among Dutch adolescents (RQ1), and to what extent latent class membership was associated with adolescent demographics (RQ2) and parental health behaviors (RQ3). Before the pandemic, three clusters were identified: one class characterized by health-risk substance use and food intake, one class reflecting a health-protective version of these behaviors, and a medium health-risk class. Most adolescents in the health-risk or health-protective class transitioned to a similar class during the pandemic phases, maintaining comparable patterns of risk or protective behaviors. The third, medium-risk, class was split into a health-protective and a health-risk class (regarding substance use and food intake) during the pandemic, with most adolescents transitioning to the health-risk class. Age, educational type, sex, and parental behavior were related to latent class membership at some, but not all, timepoints. Parental health-risk behavior -but not adolescent demographic factors- were associated with the transition from the medium to the health-risk class.

### Latent class membership and transitions

In the current study, we found three clusters based on adolescents’ health-related behaviors before the pandemic and we identified two clusters during the four examined phases of the COVID-19 pandemic. In all phases, we found a cluster that was consistently low in substance use and unhealthy food intake, which is in line with previous studies finding a health-protective cluster [16, 18]. In line with this prior work [16, 18], this was the largest class, suggesting that most adolescents in our sample maintain a generally healthy lifestyle. Furthermore, in line with previous work [15, 16, 19], we found clear links among the separate substance use health behaviors. More specifically, our findings indicate that the use of nicotine and alcohol, as well as abstention from these substances, frequently co-occurred within the same individuals. Additionally, we observed clustering within the food intake behaviors, showing that more unhealthy snacking co-occurred with less fruit and vegetable intake, and vice-versa, in line with earlier work [35, 47]. These findings also confirm the previously found pattern that health behaviors may cluster at both ends of the health-risk to health-protective spectrum [16, 48].

Beyond clustering within the same health behavior domains (i.e., substance use and food intake behaviors), we also observed clustering across domains. Across most clusters, we observed that health-risk substance use co-occurred with health-risk food intake, and that health-protective substance use co-occurred with health-protective food intake. Although this pattern has been identified previously, it could be a pattern that is particularly prominent in adolescents, as it has not been reported consistently in studies in adults [16], and when reviewing multiple developmental phases [15]. This indicates that adolescent health behaviors may be best understood as part of broader lifestyle patterns rather than isolated health habits, and that some adolescents consistently adopt health-protective behaviors, while others cluster in risk-oriented pattern. Furthermore, the inter-domain clustering between physical activity and food intake was less consistent than what is usually found in previous work [15, 16]. Interestingly, pre-pandemic health-risk substance use and food intake were associated with health-*protective* physical activity. It is possible that while in adults, increased physical activity is a protective factor for substance use and food intake [16], it may be a risk factor in youth [49–51]. Possible explanations are that participation in moderate and vigorous types of physical activity may widen an adolescent’s social network (e.g., in sport clubs), opening up opportunities to experiment with the use of substances [52] and/or that adolescents may feel like they can compensate their health-risk behaviors by engaging in health-protective physical activity [53]. Notably, during the pandemic, particularly during periods of lockdowns, health-risk substance use and food intake were no longer clustered with health-*protective* physical activity, but with health*-risk* physical activity. This may be the case because during the pandemic, opportunities to be physically active were limited, as shown by studies showing clear decreases in physical activity [11], likely regardless of other health behaviors.

Although most participants stayed in similar classes regarding substance use and food intake behavior, with continuous changes in physical activity visible due to COVID-19 measures, there were also adolescents who did change. During the first transitions, most of these changes were made by adolescents moving from the health-risk substance use and food intake class to the health-protective class, which is in line with findings from previous work during the first part of the pandemic [18]. Although we did not conduct formal significance tests, descriptively transitions appeared more frequent from (re)opening phases to lockdown phases than vice versa. This may indicate that during lockdowns, the opportunities to show health-risk behaviors might have been smaller than before, for instance due to decreased exposure to peers, decreased access to availability of substances and unhealthy food, and being restricted to stay home with, and be more closely monitored by parents [6, 12]. For the final two transitions, it seemed as if more adolescents moved from the health-protective to the health-risk substance use and food intake class; however, these differences were not tested for significance). This may indicate that as adolescents were getting older, more adolescents were likely to engage in health-risk behaviors, particularly during the (second) reopening phase when there were fewer restrictions. Further changes in the last phases of the pandemic and after the pandemic need to be examined in other studies.

### Correlates of latent class membership and transitions

Several correlates of the clusters of health behaviors were assessed as well. Regarding adolescent demographics, older adolescents, adolescents in the lower educational type, and boys had higher odds to be in the health-risk class regarding substance use and food intake or the medium-risk class than the health-protective class. Although other studies have found age to be related to lower-risk health behavior [16, 18], these studies included older youth and adults. Research indeed shows that health-risk behavior tends to be low in childhood, increases around puberty, peaks in late adolescence to early adulthood, and decreases in adulthood [54]. Our findings on educational type and sex are in line with the previous review study by Noble et al. [16]. It should be noted, though, that most correlates were only identified pre-pandemic, not during the pandemic (except for the role of age, which was also found at reopening 1). Explanations could be that as the pandemic may have reduced opportunities “across the board” to show health-risk behaviors [6], engaging in them may have become less dependent on age, sex, and educational type. It is also plausible that our large degree of participant drop-out may have resulted in a decrease in statistical power to detect associations. Hence, larger studies, across the entire pandemic period, are essential to corroborate these findings.

Besides demographics, adolescents had higher odds to be in the medium-risk cluster pre-pandemic (compared to the health-protective cluster) if their parents consumed more sugar-sweetened beverages and less fruit and vegetables. In line with previous work from our group [35, 55], this indicates that parental food consumption may be predictive of different health-risk behaviors. This study shows that the other parental health behaviors may be less predictive of adolescent health behavior clusters, although it should also be noted that food intake comprises four out of eight of the indicators, and as such forms a big part of the health behavior cluster of adolescents. As the associations were only found pre-pandemic, it is possible that as adolescents get older, parental influence diminishes. It is also possible that with adolescents spending more time at home with their parents during a period in which gaining independence is a key developmental task, the parent–child relationship and interactions may be under pressure [37]. Potential conflicts and a poor parent–child relationship quality may result in absent parent–child resemblance in health behaviors [38], even though increased exposure may usually lead to higher resemblance (van den [35]). Finally, as mentioned before, it is also likely that decreased statistical power may explain why we did not find consistent results during the pandemic.

Regarding transitions, it is notable that the third, medium-risk, class pre-pandemic was split into a health-protective and a health-risk class regarding substance use and food intake during the first lockdown. Most adolescents transitioned to the health-risk class (58%), indicating that adolescents showing some health-risk behaviors were more at-risk for unhealthy behaviors during the pandemic. Adolescent demographics were not related to this change, but it was revealed that transitioning to the health-risk class was associated with higher sugar-sweetened beverages intake and lower alcohol use by parents. Here, again, we see that a form of food intake was associated with health-risk behaviors, in line with previous work from our group [35, 55]. As alcohol use was not in the expected direction, it may have been confounded by parents’ educational type. Research shows that adults with a higher educational attainment tend to consume more alcohol [56] but may generally live a healthier lifestyle [57, 58].

Beyond the tested correlates, it is likely that class membership and transitions may be predicted by other (pre-)pandemic factors not assessed in the current study model. For instance, in their pre-print, Kiri & Brandt [28] found that a history of adverse childhood experiences could put people at risk of engaging in more risky health behaviors during the pandemic. Also during the pandemic, increased stress and poor mental health may be risk factors for health-risk behaviors. For example, Salazar-Fernández et al. [18] found that people in the higher-risk clusters were affected more and reported higher levels of stress, anxiety, depression, and loneliness than those in the lower-risk cluster. Taken together, future studies could assess other correlates of class membership and transitions thereof.

### Strengths and limitations

This study had several strengths. First, we used a person-centered approach that acknowledges the differential effects of the pandemic on health behaviors over time [25–27]. Second, we were able to capture many health behaviors simultaneously in one analysis. Hence, we were able to capture the most relevant modifiable risk factors for developing non-communicable diseases [14], including physical activity, which was not measured in previous similar work [18, 28]. Methodologically, entering multiple health behaviors into one model simultaneously reduced the likelihood of type 1 error by eliminating the need to analyze each health behavior independently [59]. Third, we used a prospective research design with data available from both before and different phases of the pandemic.

Of course, there are also limitations to this study. First, it should be mentioned that the current findings might specifically apply to the situation in the Netherlands, as COVID-19 measures differed significantly across countries (and even regions) depending on infection numbers and the vision of policy makers [60]. Second, attrition was substantial over time, particularly from pre-pandemic to the first lockdown. We estimated models using FIML, under a Missing At Random (MAR) assumption, which limits bias relative to listwise deletion. However, FIML does not address MNAR (Missing Not At Random) mechanisms, thus selective dropout remains an important limitation. Accordingly, our findings should be interpreted with caution and replicated in more diverse Dutch samples (including lower-educated ones), and in other countries. Third, and related to the second, as we treated all variables as categorical, it is likely that there were only few participants in certain categories (e.g., of the food intake variables with 8 categories) of certain latent classes. Increasing sample size with sufficient variability across categories is warranted. Fourth, it should be acknowledged that decision-making and labeling of the classes in the LTA is, to some extent, subjective. We aimed to reduce this subjectivity by first making independent classifications by three different researchers and reaching consensus. Fifth, as we did not measure light physical activity (i.e., walking) at all waves, we could not include it in our measure of physical activity. However, as walking was a popular activity during the pandemic in the Netherlands [61], this would have been a relevant indicator. Sixth and seventh, we used self-reports of all health behaviors and the health behaviors during the first lockdown were assessed retrospectively, which can introduce biases, such as social desirability bias and recall bias [62]. Eight, as the LTA could not include covariates with missings, we had to use (multinomial) logistic regressions in R. It is preferable to include all covariates in the LTA in a future study.

## Conclusions

Taken together, we found that adolescents’ health behaviors meaningfully cluster in latent classes before and during the COVID-19 pandemic. As interventions currently mostly focus on one health-risk behavior, multiple behaviors should be considered when developing new interventions, resulting in more integrative intervention approaches. We also found that adolescents who start in a health-protective class, usually stay in a health-protective class throughout the pandemic, and adolescents who start in a health-risk class, usually stay in a health-risk class throughout the pandemic. This shows that health behavior is relatively stable even through different phases of the pandemic. Before the pandemic, a third, medium-risk, class was found that was split into a health-protective and a health-risk class (regarding substance use and food intake) during the pandemic, with most adolescents transitioning to the health-risk class. This may indicate that during the pandemic, behaviors were more extreme. It should be further assessed whether this finding generalizes to stressful times beyond the COVID-19 pandemic. Finally, our results suggest that adolescent demographics and parental food intake behaviors are relevant to consider as forms of screening adolescents who may be more at risk of showing health-risk behaviors. Larger studies, including different pre-, during-, and post-pandemic phases are needed to corroborate these findings.

## Supplementary Information

Supplementary Material 1.

Supplementary Material 2.

## Data Availability

Our R scripts, Mplus Input Files, and Mplus Output Files can be found on the Open Science Framework (https://osf.io/8yznm/files/). The analytic datasets supporting the conclusions of this article are available on the Radboud Data Repository (https://doi.org/10.34973/eekd-9v39).
