# Supplementary material for: Clustering of adolescents’ health behaviors before and during the COVID-19 pandemic: examining transitions and the role of demographics and parental health behaviors
Source: BMC Public Health. 2025 Dec 6;26:149. doi: 10.1186/s12889-025-25368-3 (PMC12797725; doi:10.1186/s12889-025-25368-3)
Supplement: Supplementary file 1 — Supplementary Material 1. [file 12889_2025_25368_MOESM1_ESM.docx]

**SUPPLEMENTAL FILE**

**Clustering of Adolescents’ Health Behaviors Before and During the COVID-19 Pandemic: The Role of Demographics and Parental Health Behaviors**

Nina van den Broek, Linnea Cederlund, Emma Koenders, Renske van der Cruijsen,

Junilla K. Larsen, Roy Otten, & Jacqueline M. Vink

| Topic | Text | Table | Figure | Page(s) |
| --- | --- | --- | --- | --- |
| Deviations from Pre-Registration | A |  |  | 2 |
| LCA Results Parents |  | A |  | 3 |
| Timeline of the Research Project |  |  | A | 4 |
| Analytical Sample Size Across Study Waves |  | B |  | 5 |
| Attrition Analysis |  | C |  | 6 |
| Detailed Measures Information | B |  |  | 7 - 12 |
| Additional Details Data-Analyses Steps | C |  |  | 13 - 14 |
| References Supplemental Text | D |  |  | 15 - 16 |
| Descriptive Statistics of Adolescents’ Health Behaviors |  | D |  | 17 - 18 |
| Correlations Between Adolescents’ Health Behaviors and Demographics |  | E |  | 19 |
| Odds Ratios Demographics Covariates Predicting Adolescent Class Membership |  | F |  | 20 |
| (Multinomial) Logistic Regressions of Parental Health Behaviors Predicting Adolescent Class Membership |  | G to K |  | 21 - 25 |

**Supplemental Text A: Deviations from Pre-Registration**

In the current study, we made several deviations from our pre-registration. *First*, we pre-registered to not only perform an LTA on the adolescent health behaviors, but also on the parental health behaviors. However, this turned out to be infeasible, given that for parents, the 1-class solution was found to be most optimal across timepoints (see Supplemental Table A). Therefore, instead of linking class membership of parents and adolescents, we investigated the effect of the eight parental health behaviors indicator on adolescents’ class membership for the different waves using (multinomial) logistic regressions to address the third research question. *Second*, in all models, we used the MLR estimator, not the WLSMV as preregistered, since WLSMV did not seem to work with mixture models in Mplus. *Third*, in Step 4 of the LTA, we tried to include the three demographic covariates (i.e., age, educational type, and sex) in the LTA model, as pre-registered. As we experienced estimation issues due to data sparsity when the interaction terms (effects on transition probabilities) were added, we only examined main effects of the covariates (effects on class membership). *Fourth*, a couple of additional (exploratory) analyses were preregistered, but not performed, as they would run beyond the scope of this article. Specifically, we preregistered to examine the moderating influence of relationship satisfaction on similarity in clustering of parental and adolescent health behaviors, but did not do so given that we did not find different parental latent classes. *Fifth*, the exploratory analysis using light physical activity was not performed given that this information was not available for all waves, complicating comparisons with the main analyses. *Sixth* and finally, sensitivity analyses using complete cases and using imputed datasets were not performed due to high complexity of the analyses as it is.

**Supplemental Table A.**

*Fit Indices for Different Number of Classes Across the Five Timepoints for Parents.*

| # classes | Par | LL | AIC | aBIC | VLMR-LRT (*p*) | BLRT (*p*) | Entropy | Percentage smallest class | Average class assignment probability per class | | | | | | | | |
| --- | --- | --- | --- | --- | --- | --- | --- | --- | --- | --- | --- | --- | --- | --- | --- | --- | --- |
| Pre-pandemic |  |  |  |  |  |  |  |  |  | | | | | | | | |
| 1 | 36 | -3,501.92 | 7,075.84 | 7,110.85 | n.a. | n.a. | n.a. | n.a. | n.a. | | | | | | | | |
| 2 | 73 | -3,464.03 | 7,074.05 | 7,145.05 | .604 | .008 | .53 | 30% | .88 | | | | | .77 | | | |
| 3 | 110 | -3,431.43 | 7,082.86 | 7,189.84 | .797 | .065 | .63 | 8% | .81 | | .77 | | | | | .80 | |
| 4 | Did not converge | | | | | | | | | | | | | | | | |
| Lockdown 1 |  |  |  |  |  |  |  |  |  | | | | | | | | |
| 1 | 36 | -2,604.54 | 5,281.07 | 5,304.40 | n.a. | n.a. | n.a. | n.a. | n.a. | | | | | | | | |
| 2 | 73 | -2,569.30 | 5,284.60 | 5,331.90 | .827 | .009 | .45 | 49% | .82 | | | | .83 | | | | |
| 3 | 110 | -2,537.08 | 5,294.16 | 5,365.44 | .764 | .030 | .68 | 8% | .85 | | | .83 | | | .81 | | |
| 4 | 147 | -2,511.09 | 5,316.18 | 5,411.43 | .847 | .336 | .73 | 9% | .82 | .96 | | | .89 | | | | .83 |
| Reopening 1 |  |  |  |  |  |  |  |  |  | | | | | | | | |
| 1 | 36 | -2,586.49 | 5,244.99 | 5,268.31 | n.a. | n.a. | n.a. | n.a. | n.a. | | | | | | | | |
| 2 | 73 | -2,549.04 | 5,244.09 | 5,291.39 | .142 | .004 | .53 | 47% | .84 | | | | .85 | | | | |
| 3 | 110 | -2,520.83 | 5,261.66 | 5,332.94 | .765 | .181 | .63 | 23% | .78 | | | .84 | | | .81 | | |
| 4 | Did not converge | | | | | | | | | | | | | | | | |
| Lockdown 2 |  |  |  |  |  |  |  |  |  | | | | | | | | |
| 1 | 36 | -1,776.88 | 3,625.76 | 3,634.64 | n.a. | n.a. | n.a. | n.a. | n.a. | | | | | | | | |
| 2 | 73 | -1,746.18 | 3,638.35 | 3,656.38 | 1.000 | .078 | .96 | 13% | .93 | | | | .99 | | | | |
| 3 | 110 | -1,721.96 | 3,663.93 | 3,691.09 | .771 | .402 | .91 | 6% | .88 | | | .88 | | | .98 | | |
| 4 | Did not converge | | | | | | | | | | | | | | | | |
| Reopening 2 |  |  |  |  |  |  |  |  |  | | | | | | | | |
| 1 | 36 | -1,816.89 | 3,705.78 | 3,715.45 | n.a. | n.a. | n.a. | n.a. | n.a. | | | | | | | | |
| 2 | 73 | -1,790.36 | 3,726.71 | 3,746.33 | .956 | .263 | .68 | 25% | .86 | | | | .91 | | | | |
| 3 | Did not converge | | | | | | | | | | | | | | | | |
| 4 | Did not converge | | | | | | | | | | | | | | | | |

*Note.* Par = Number of free parameters; LL = Loglikelihood value; AIC = Akaike Information Criterion; aBIC = Sample-Size Adjusted Bayesian Information Criterion; VLMR-LRT = Vuong-Lo-Mendell-Rubin Likelihood Ratio Test; BLRT = Bootstrapped Likelihood Ratio Test. Selected number of classes are highlighted in grey. n.a. = not applicable.

**Supplemental Figure A.**

*Timeline of the “G(F)OOD together” Research Project. W = Wave. Retro = Retrospective. Examples of COVID-19 measures in the Netherlands are presented.*

***
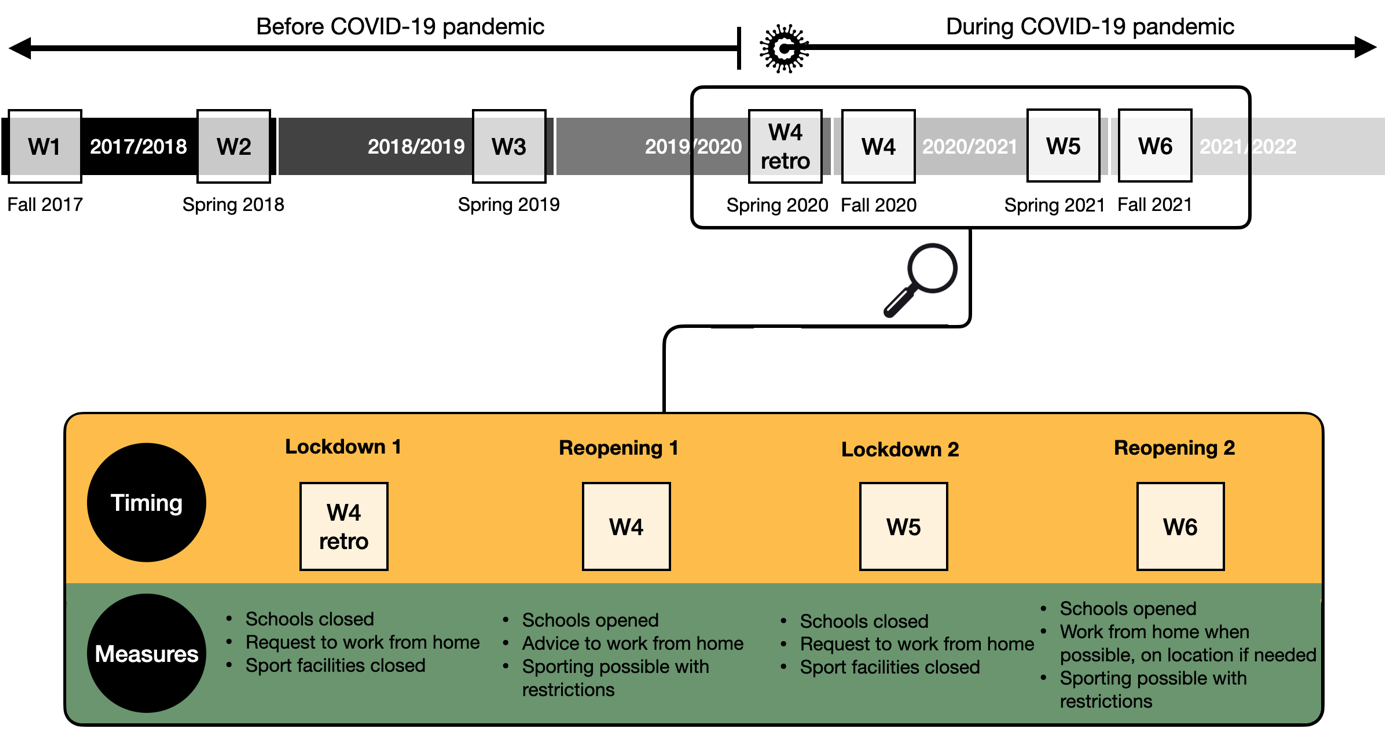
*Supplemental Table B.**

*Sample Size of the Adolescents, Mothers, and Fathers Included in the Current Study, Across the Study Waves.*

|  | Adolescents | Biological mothers | Biological fathers |
| --- | --- | --- | --- |
| Wave 3 | 674 | 303 | 110 |
| Wave 4 (retro) | 303 | 210 | 84 |
| Wave 5 | 138 | 141 | 46 |
| Wave 6 | 128 | 134 | 47 |
| Total unique | 710 | 328 | 121 |

**Supplemental Table C**

*Standardized Estimates and Significance Statistics of Main Study Variables at Pre-Pandemic Predicting Missingness After Pandemic (Attrition Analysis).*

|  | *b* | *SE* | *OR* | *p* | 95% CI | |
| --- | --- | --- | --- | --- | --- | --- |
| Age^a^ | **0.39** | **0.16** | **1.45** | **.008** | **1.09** | **2.01** |
| Educational type^b^ | -0.05 | 0.20 | 0.95 | .932 | 0.65 | 1.39 |
| Sex^c^ | 0.01 | 0.18 | 1.00 | .994 | 0.71 | 1.42 |
| Cigarette use^d^ |  |  |  |  |  |  |
| Experimental | -0.09 | 0.39 | 0.91 | .818 | 0.42 | 2.00 |
| Infrequent current | 0.61 | 1.20 | 1.84 | .609 | 0.22 | 39.14 |
| Frequent current | -0.30 | 0.93 | 0.74 | .750 | 0.13 | 5.94 |
| E-cigarette use^e^ | 0.11 | 0.51 | 1.12 | .830 | 0.42 | 3.14 |
| Alcohol use^f^ |  |  |  |  |  |  |
| Experimental | 0.15 | 0.19 | 1.16 | .442 | 0.80 | 1.68 |
| Infrequent current | **0.76** | **0.38** | **2.13** | **.045** | **1.03** | **4.55** |
| Frequent current | 1.15 | 0.88 | 3.16 | .194 | 0.64 | 23.91 |
| SSBs intake (continuous) | -0.04 | 0.04 | 0.96 | .392 | 0.89 | 1.05 |
| Sweet snack intake (continuous) | 0.02 | 0.04 | 1.02 | .685 | 0.93 | 1.12 |
| Savory snack intake (continuous) | 0.12 | 0.12 | 1.13 | .313 | 0.89 | 1.43 |
| Fruit & veg intake (continuous) | -0.07 | 0.07 | 0.93 | .342 | 0.80 | 1.08 |
| Physical activity^g^ | 0.18 | 0.19 | 1.20 | .345 | 0.82 | 1.75 |

*Note.* Statistically significant parameters (*p* < .05) are denoted in bold. OR = Odds Ratio. CI = Confidence Interval*.* ^a^Age at the date of participation in Wave 3. ^b^0 = Low educational type; 1 = High educational type. ^c^0 = male; 1 = female. ^d^Reference level for cigarette use was non-smoking. ^e^Reference level for e-cigarette use was non-use. ^f^Reference level for alcohol use was non-drinking. ^g^Effect of sufficiently active versus insufficiently active.

**Supplemental Text B: Detailed Measures Information**

***Nicotine Use***

For all participants, cigarette use was measured with one item (based on Mies et al., 2017): “Which statement about smoking applies most to you?”. A 6-point scale was used: 1: I have never smoked, not even a puff, 2: I have taken a puff of a cigarette a few times, 3: I used to smoke (regularly), but I have quit smoking now, 4: I smoke once a week or less, 5: I smoke several times a week, 6: I smoke daily. We recoded the variable so that it represents current smoking use for both adolescents and parents. Participants choosing answer option 1 and 3 were classified as “Non-Smokers”, 2 as “Experimental Smokers”, 4 as “Infrequent Current Smokers”, and 5 and 6 as “Frequent Current Smokers”.

For all participants, e-cigarette use was measured with one item, asking the participants to indicate how often they use e-cigarette. In Wave 3, participants were asked to report their e-cigarette use in the past year; in Wave 4 retro and Wave 4, they reported on the time period during lockdown and since the start of school year, respectively; in Wave 5, since the second lockdown; in Wave 6, in the past six months. For Wave 3, the response options were 0, 1, 2, 3, 4, 5, 6, 7, 8, 9, 10, 11 to 19, 20 to 39, and 40+ (based on TR&NDS study, e.g., Mies et al., 2017). For Wave 4 to 6 (including Wave 4 retro), the response options were changed to “Never”, “Sometimes”, “Regularly” (as developed by the research team). We recoded the variable so that it represents use for both adolescents and parents. Participants choosing answer option 0 in Wave 3 or Never in Waves 4 retro to 6 were classified as “Non-Users”. All other participants were classified as “Users”.

***Alcohol Use***

For all participants, alcohol use was measured with one item (based on TR&NDS study, e.g., Mies et al., 2017). Participants were asked to indicate a response to the question: “Which statement about alcohol applies most to you?” on a 6-point scale: 1: I have never drunk alcohol, not even a sip, 2: I have had a sip of alcohol a few times, 3: I used to drink alcohol (regularly), but I don't drink anymore, 4: I drink alcohol once a week or less, 5: I drink alcohol several times a week, 6: I drink alcohol daily. We recoded the variable so that it represents current alcohol use for both adolescents and parents. Participants choosing answer option 1 and 3 were classified as “Non-Drinkers”, 2 as “Experimental Drinkers”, 4 as “Infrequent Current Drinkers”, and 5 and 6 as “Frequent Current Drinkers”.

***Food Intake***

Food intake was measured at all waves using a food frequency questionnaire (FFQ). Participants were asked how frequently they consume certain foods and beverages. More specifically, the intake of eight items were assessed: (1) soft drinks (carbonated and non-carbonated drinks with sugar, diet drinks excluded; e.g., cola or lemonade); (2) cake, pastry, and large cookies (e.g., donut or muffin); (3) candy bars (e.g., chocolate-covered bars or confections); (4) chocolate (e.g., chocolate bars or pralines); (5) warm, fried snacks (e.g., sausage roll or pizza slice); (6) fruit (e.g., apple or banana); (7) salad and raw vegetables (e.g., cherry tomatoes or cucumber); and (8) heated vegetables (i.e., cooked, baked, steamed, or otherwise heated; e.g., broccoli or green beans). Participants were asked to indicate their intake of each item on an 8-point scale ranging from ‘0 days a week’ (0) to ‘7 days a week’ (7). Our rationale for these items is presented in our previous work (van den Broek et al., 2020) The questions differed for adolescents and parents, and for adolescents also between waves. For Waves 3, 4 retro and 4, adolescents were asked to indicate how many days per week they obtained the specific food and beverage items in four different contexts: (1) taken or received from home, to eat or to drink at home or to take away; (2) bought at school, such as from the canteen or the vending machine; (3) bought somewhere else, such as in the supermarket, snack bar, or sports club; and (4) received somewhere else, such as at their neighbors’, grandparents’, or friends’ place. For Waves 5 and 6, adolescents were only asked for total consumption of the food and beverage items per week (no context). Parents were asked at each wave to report how many days per week they consumed the specific food and beverage items in both the presence and absence of their child.

The eight FFQ items were combined to form four indices: sugar-sweetened beverages (SSBs), sweet snacks, savory snacks, and fruit and vegetables (van den Broek et al., 2020). Scores for soft drinks (item 1 of the FFQ) were used to obtain the measure for SSBs. Scores for cake, candy bars, and chocolate (items 2–4 of the FFQ) were used to assess sweet snacks. Scores for warm, fried snacks (item 5 of the FFQ) were used to assess savory snacks. Scores for fruit, salad and raw vegetables, and heated vegetables (items 6–8 of the FFQ) were used to assess fruit and vegetables (for more detailed information, see the Transformations section). The items measuring sweet and savory snack intake have been selected from a validated Dutch FFQ measuring fat intake (van Assema et al., 2001). In line with previous studies (van den Broek et al., 2018, 2020; Wouters et al., 2010), all items assessing the intake of sweet snacks were included. However, some modifications were made with regard to the items assessing savory snack intake (van Assema et al., 2001). We disregarded two items on “nuts and peanuts” and on “potato chips, pieces of cheese and sausage”, since the Dutch Nutrition Centre considers (low-fat) cheese and (unsalted) nuts to be part of a healthy diet. The items to assess fruit and vegetable intake were also selected from a validated Dutch FFQ (van Assema et al., 2002)The only item that was disregarded was the item of fruit juice, as the Dutch Nutrition Centre does not advise to consume fruit juices instead of whole fruit. Juice is considered a drink high in sugar, providing a lot of calories, and is associated with overweight and diabetes type 2 (Brink et al., 2019).

To categorize food intake, a maximum of days per week was determined, ranging from 0 to 7 days, for each of the four indices (SSBs, sweet snacks, savory snacks, fruit and vegetables). The steps conducted to determine the maximum varied between adolescents and parents, waves, and indices. For adolescents, in Wave 3, Wave 4 retro and Wave 4, adolescents were asked to report their food intake separately for four different contexts: (1) taken or received from home, to eat or to drink at home or to take away; (2) bought at school, such as from the canteen or the vending machine; (3) bought somewhere else, such as in the supermarket, snack bar, or sports club; and (4) received somewhere else, such as at their neighbors’, grandparents’, or friends’ place. For these waves, the first step was to identify and select the highest value reported across the different contexts for each food intake variable. For example, if cake intake was reported as 5 days per week for context (1), but 2 days per week in the other contexts, the maximum value for cake was identified as 5 days per week.

For parents, in all waves, parents were asked to report their food intake in two contexts: (1) in the presence of the adolescent and (2) in the absence of the adolescent. To calculate the maximum days per week, the first step was to identify and select the highest value from these contexts for each food intake variable comprising the four indices.

For sweet snacks and fruit and vegetables, as they comprised of more than one food or beverage item, the maximum weekly value for the food variables that were combined to form the indices was taken. For example, the sweet snacks index consists of the items cake, chocolate and candy bars. Thus, the maximum of days per week for sweet snacks was determined by comparing the highest values in each of the three items and selecting the highest one. For instance, if 5 days per week was reported for cake, and 2 days and 4 days for the other two variables respectively, then 5 days per week was determined as the maximum of days per week for the sweet snacks index. Consequently, this resulted in one maximum score per indicator (SSBs, sweet snacks, savory snacks, fruit and vegetables) per participant. This score indicates the highest number of days that the participant reported for any food or beverage item in any context of that index.

***Physical Activity***

Physical activity was measured using the short version of the International Physical Activity Questionnaire (IPAQ, Craig et al., 2003). Participants were asked to indicate the frequency (0-7 days per week) and amount (hours or minutes per day) for walking (walking at school and home, walking to get from one place to another, and all other walking during sports or leisure time), moderate activity (activities that make you breathe slightly faster than normal, such as regular cycling or doubles tennis) and vigorous activity (activities that require a lot of effort and cause much faster breathing, such as hockey, fast cycling or tennis). We did not focus on walking (light physical activity) in the current study. Parents were not asked to report on their physical activity in Wave 3. Therefore, we used their data from Wave 2 as a proxy for Wave 3. Previous research has suggested that physical activity in adults is stable over time, when corrected for seasonal changes (Bree et al., 2017; Martins et al., 2017). The time period between Wave 2 and Wave 3 is approximately 1 year, and both waves of data collection took place in Spring. Both waves were before the COVID-19 pandemic. Therefore, it can be reasonably assumed that physical activity scores for parents in Wave 2 can be used as a proxy for physical activity of parents in Wave 3.

To dichotomize physical activity, we have conducted several steps of recoding. More specifically, for Waves 4 retro, 4, 5 and 6, participants were asked to indicate the amount of time they spent on each physical activity in hours, and all these responses were recoded into minutes. More specifically, daily average duration of physical activity was assessed on a seven-point scale, ranging from 0 (“0 h per day”) to 6 (”3 or more hours per day”) in case they performed the corresponding type of physical activity at least once a week. We then converted the categorical variable to a numeric format ranging from 0 to 180, representing the total minutes participants engaged into a physical activity (e.g., 0 = 0; 1 = 30 min; 2 = 60 min; 3 = 90 min). For Wave 3 for adolescents and Wave 2 for parents, participants could indicate the amount of time spent on each physical activity in hours as well as minutes per week in writing their answers down in a free, numeric format (resulting in two separate variables for these waves). The variables were merged, creating a minutes per day variable for each physical activity (moderate activity and vigorous activity). Furthermore, in accordance with the guidelines of IPAQ Research Committee (2005) the following was done for all waves: If a participant had reported less than 10 minutes per day for one of the activities (walking, moderate activity and vigorous activity), we recoded the number of minutes per day for that activity to 0. For each physical activity, the amount of time spent was truncated to be equal to 180 minutes. Total minutes of physical activity of each intensity category was computed by multiplying the daily average minutes of MPA and VPA by the corresponding intensity days.

Physical activity was categorized as dichotomous (sufficiently active/insufficiently active). Based on Dutch guidelines for physical activity, adolescents and parents were categorized as sufficiently active if they spent at least 420 minutes per week or at least 150 minutes per week on moderate/vigorous physical activity, respectively (Health Council of the Netherlands, 2017). Adolescents and parents with physical activity levels that did not reach the respective cut-offs, were categorized as insufficiently active.

***Demographics***

*Sex*: Adolescents were asked to indicate their sex and the response options were “Boy” or “Girl”. *Age*: Age of adolescents was computed based on their indicated day of birth and date of participation at Wave 3. If adolescents did not participate in Wave 3 (i.e., had a missing value on date of participation), the mean age of the participating adolescents was imputed so an age could be computed for all participants. Adolescents’ *educational type* was assessed by the researcher based on information from the school and categorized as (0) pre-vocational training (in Dutch: “vmbo”) and (1) higher general or pre-university training (in Dutch: “havo/vwo”). 7 were recoded into the best-fitting category based on their textual response.

**Supplemental Text C: Additional Details Data-Analyses Steps**

***Step 0: Descriptive Statistics***

No additional details to report.

***Step 1: Study Measurement Model Alternatives for Each Time Point***

For the AIC and aBIC, lower values indicate higher model fit. For the VLMR-LRT and BLRT, a significant *p*-value (*p* < .05) indicates that the current model fits significantly better than the model with one class less. Furthermore, we looked at classification diagnostics for models with the best fit (Sorgente et al., 2019). Specifically, we looked at entropy, which is a measure of quality of class assignment (should be preferably higher than .70; Fonseca & Cardoso, 2007). As very high entropy can indicate overfitting (Sinha et al., 2021), we also took interpretability into account when choosing the number of classes. Additionally, we examined another measure of quality of class assignment, Average class assignment probabilities, which preferably should be higher than .80 (Geiser, n.d.).

If relative fit criteria and model diagnostics pointed to two or more different number of classes, we chose the most parsimonious model that is well interpretable and makes theoretical sense and does not have classes of very small size (i.e., < 5% of participants in one class; Nylund-Gibson & Choi, 2018). After fitting the models, three researchers of the research team (i.e., NB, LC, and EK) independently selected their best-fitting class solution and discussed their insights based on the fit indices and diagnostic criteria.

***Step 2: Explore Transitions Based on Cross-Sectional Results and Measurement Invariance***

To assess measurement invariance, we inspected whether the item-response probabilities were similar across waves. If so, we compared whether a measurement model that is fully invariant across time (i.e., measurement parameters constrained to be equal across time) fitted equally well as a model that is fully variant across time (i.e., measurement parameters unconstrained across time). The measurement parameters correspond to the item-response probabilities of the health behavior indicators of the classes at each wave (Nylund, 2007). We used a likelihood ratio test based on loglikelihood values and scaling correction factors obtained with the MLR estimator. If there was full measurement invariance, the characteristics of the classes (i.e., item-response probabilities of the health behavior indicators) were the same across the waves and they could be directly compared with each other. If we could not assume full measurement invariance, we aimed to proceed by releasing parameters until we obtained a model that fit equally well as the baseline model. As advised by Perra (2020), we did not impose a structure to the associations between classes yet in this step (i.e., we avoided regressing latent classes at one time point on latent classes at the previous time point).

***Step 3: Explore Specifications of the LTA without Covariates***

We used the three-step approach introduced by Vermunt (2010), and further (practically) explained by other scholars (Asparouhov & Muthén, 2014; Nylund-Gibson et al., 2014; Perra, 2023; Sorgente et al., 2019), as this approach has been shown to prevent a shift in the measurement model of the LCA model when introducing structural elements (Asparouhov & Muthén, 2014). In the first step, the most likely measurement model was estimated, as already done in Step 1 of our analytic approach. In the second step, the most likely latent class variable is created using the latent class posterior distribution obtained during the first step, as already done in Step 2 of our analytic approach. In the third step, the actual LTA is performed (i.e., adding the autoregressive paths between the latent class variables), considering the misclassification defined in the second step. This misclassification information was obtained in Mplus under the logit values for the classification probabilities.

***Step 4: Include Covariates in the LTA Model***

No additional details to report.

**Supplemental Text D: References Supplemental Text**

Asparouhov, T., & Muthén, B. (2014). Auxiliary Variables in Mixture Modeling: Three-Step Approaches Using Mplus. *Structural Equation Modeling: A Multidisciplinary Journal*, *21*(3), 329-341. https://doi.org/10.1080/10705511.2014.915181

Bree, R. J. H. van, Bolman, C., Mudde, A. N., Stralen, M. M. van, Peels, D. A., Vries, H. de, & Lechner, L. (2017). Modeling Longitudinal Relationships Between Habit and Physical Activity: Two Cross-Lagged Panel Design Studies in Older Adults. *Journal of Aging and Physical Activity*, *25*(3), 464-473. https://doi.org/10.1123/japa.2016-0212

Brink, E., Van Rossum, C., Postma-Smeets, A., Stafleu, A., Wolvers, D., Van Dooren, C., Toxopeus, I., Buurma-Rethans, E., Geurts, M., & Ocké, M. (2019). Development of healthy and sustainable food-based dietary guidelines for the Netherlands. *Public Health Nutrition*, *22*, 2419-2435. https://doi.org/10.1017/S1368980019001435

Craig, C. L., Marshall, A. L., Sjöström, M., Bauman, A. E., Booth, M. L., Ainsworth, B. E., Pratt, M., Ekelund, U., Yngve, A., Sallis, J. F., & Oja, P. (2003). International Physical Activity Questionnaire: 12-country reliability and validity. *Medicine & Science in Sports & Exercise*, *35*, 1381-1395. https://doi.org/10.1249/01.mss.0000078924.61453.fb

Fonseca, J. R. S., & Cardoso, M. G. M. S. (2007). Mixture-model cluster analysis using information theoretical criteria. *Intelligent Data Analysis*, *11*(2), 155-173. https://doi.org/10.3233/IDA-2007-11204

Geiser, C. (z.d.). *Latent transition analysis with Mplus*. QuantFish. https://www.goquantfish.com/courses/take/latent-transition-analysis-with-mplus/lessons/43100590-model-fit-part-3-model-comparisons

Martins, R. C., Reichert, F. F., Bielemann, R. M., & Hallal, P. C. (2017). One-year Stability of Objectively Measured Physical Activity in Young Brazilian Adults. *Journal of Physical Activity and Health*, *14*(3), 208-212. https://doi.org/10.1123/jpah.2015-0384

Mies, G. W., Treur, J. L., Larsen, J. K., Halberstadt, J., Pasman, J. A., & Vink, J. M. (2017). The prevalence of food addiction in a large sample of adolescents and its association with addictive substances. *Appetite*, *118*, 97-105. https://doi.org/10.1016/j.appet.2017.08.002

Nylund, K. L. (2007). *Latent Transition Analysis: Modeling Extensions and an Application to Peer Victimization* [University of California]. http://www.statmodel.com/download/Nylund%20dissertation%20Updated1.pdf

Nylund-Gibson, K., & Choi, A. Y. (2018). Ten frequently asked questions about latent class analysis. *Translational Issues in Psychological Science*, *4*(4), 440-461. https://doi.org/10.1037/tps0000176

Nylund-Gibson, K., Grimm, R., Quirk, M., & Furlong, M. (2014). A Latent Transition Mixture Model Using the Three-Step Specification. *Structural Equation Modeling: A Multidisciplinary Journal*, *21*(3), 439-454. https://doi.org/10.1080/10705511.2014.915375

Perra, O. (2020). *Latent transition analysis*. SAGE Research Methods: Foundations.

Perra, O. (2023). *Introduction to Latent Transition Analysis. National Centre for Research Methods online learning resource.* https://www.ncrm.ac.uk/resources/online/all/?id=20821

Sinha, P., Calfee, C. S., & Delucchi, K. L. (2021). Practitioner’s Guide to Latent Class Analysis: Methodological Considerations and Common Pitfalls. *Critical Care Medicine*, *49*, e63-e79.

Sorgente, A., Lanz, M., Serido, J., Tagliabue, S., & Shim, S. (2019). Latent transition analysis: Guidelines and an application to emerging adults’ social development. *TPM - Testing*, *26*, 39-72. https://doi.org/10.4473/TPM26.1.3

van Assema, P., Brug, J., Ronda, G., & Steenhuis, I. (2001). The relative validity of a short Dutch questionnaire as a means to categorize adults and adolescents to total and saturated fat intake. *Journal of Human Nutrition and Dietetics*, *14*, 377-390. https://doi.org/10.1046/j.1365-277X.2001.00310.x

van Assema, P., Brug, J., Ronda, G., Steenhuis, I., & Oenema, A. (2002). A short dutch questionnaire to measure fruit and vegetable intake: Relative validity among adults and adolescents. *Nutrition and Health*, *16*, 85-106. https://doi.org/10.1177/026010600201600203

van den Broek, N., Larsen, J. K., Verhagen, M., Burk, W. J., & Vink, J. M. (2020). Is adolescents’ food intake associated with exposure to the food intake of their mothers and best friends? *Nutrients*, *12*, 786-786. https://doi.org/10.3390/nu12030786

van den Broek, N., Larsen, J. K., Verhagen, M., Eisinga, R., Burk, W. J., & Vink, J. M. (2018). The longitudinal link between mothers’ and adolescents’ snacking: The moderating role of television viewing. *Appetite*, *120*, 565-570. https://doi.org/10.1016/j.appet.2017.10.010

Vermunt, J. K. (2010). Latent Class Modeling with Covariates: Two Improved Three-Step Approaches. *Political Analysis*, *18*(4), 450-469. https://doi.org/10.1093/pan/mpq025

Wouters, E. J., Larsen, J. K., Kremers, S. P., Dagnelie, P. C., & Geenen, R. (2010). Peer influence on snacking behavior in adolescence. *Appetite*, *55*, 11-17. https://doi.org/10.1016/j.appet.2010.03.002

**Supplemental Table D.**

*Descriptive Statistics (Valid N and % of Participants in Each Indicator Category) of Adolescents’ Health Behaviors.*

| Health Behavior Indicator | Valid *N* | Non-smokers | Experimental smokers | Infrequent smokers | Frequent smokers |  |  |  |  |
| --- | --- | --- | --- | --- | --- | --- | --- | --- | --- |
| Cigarette use |  |  |  |  |  |  |  |  |  |
| Pre-pandemic | 672 | 89.4% | 7.9% | 1.5% | 1.2% |  |  |  |  |
| Lockdown 1 | 271 | 87.8% | 8.9% | 0.7% | 2.6% |  |  |  |  |
| Reopening 1 | 272 | 88.2% | 7.7% | 1.5% | 2.6% |  |  |  |  |
| Lockdown 2 | 138 | 82.6% | 15.2% | 2.2% | 0.0% |  |  |  |  |
| Reopening 2 | 128 | 77.3% | 18.0% | 3.1% | 1.6% |  |  |  |  |
|  | Valid *N* | Non-users | Users |  |  |  |  |  |  |
| E-cigarette use |  |  |  |  |  |  |  |  |  |
| Pre-pandemic | 671 | 94.3% | 5.7% |  |  |  |  |  |  |
| Lockdown 1 | 279 | 96.8% | 3.2% |  |  |  |  |  |  |
| Reopening 1 | 279 | 96.4% | 3.6% |  |  |  |  |  |  |
| Lockdown 2 | 138 | 94.2% | 5.8% |  |  |  |  |  |  |
| Reopening 2 | 128 | 95.3% | 4.7% |  |  |  |  |  |  |
|  | Valid *N* | Non-drinkers | Experimental drinkers | Infrequent drinkers | Frequent drinkers |  |  |  |  |
| Alcohol use |  |  |  |  |  |  |  |  |  |
| Pre-pandemic | 671 | 37.7% | 48.3% | 12.1% | 1.9% |  |  |  |  |
| Lockdown 1 | 277 | 44.8% | 24.2% | 20.6% | 10.5% |  |  |  |  |
| Reopening 1 | 276 | 41.3% | 26.4% | 25.4% | 6.9% |  |  |  |  |
| Lockdown 2 | 138 | 34.1% | 28.3% | 31.9% | 5.8% |  |  |  |  |
| Reopening 2 | 128 | 28.9% | 23.4% | 34.4% | 13.3% |  |  |  |  |
|  | Valid *N* | 0 times per week | 1 time per week | 2 times per week | 3 times per week | 4 times per week | 5 times per week | 6 times per week | 7 times per week |
| SSBs intake |  |  |  |  |  |  |  |  |  |
| Pre-pandemic | 674 | 7.4% | 16.9% | 23.9% | 16.3% | 9.3% | 7.1% | 3.9% | 15.1% |
| Lockdown 1 | 303 | 14.5% | 19.1% | 14.5% | 14.2% | 7.3% | 6.6% | 3.3% | 20.5% |
| Reopening 1 | 303 | 11.2% | 22.1% | 19.5% | 11.9% | 7.6% | 7.9% | 3.3% | 16.5% |
| Lockdown 2 | 138 | 18.8% | 19.6% | 18.1% | 12.3% | 2.9% | 5.1% | 5.8% | 17.4% |
| Reopening 2 | 128 | 13.3% | 27.3% | 23.4% | 7.8% | 7.0% | 5.5% | 2.3% | 13.3% |
|  | Valid *N* | 0 times per week | 1 time per week | 2 times per week | 3 times per week | 4 times per week | 5 times per week | 6 times per week | 7 times per week |
| Sweet snacks intake |  |  |  |  |  |  |  |  |  |
| Pre-pandemic | 674 | 2.4% | 16.6% | 26.4% | 18.4% | 11.0% | 9.9% | 4.3% | 11.0% |
| Lockdown 1 | 302 | 3.3% | 11.9% | 21.9% | 14.6% | 11.9% | 11.9% | 4.6% | 19.9% |
| Reopening 1 | 302 | 2.6% | 13.2% | 28.5% | 14.2% | 7.3% | 12.3% | 5.0% | 16.9% |
| Lockdown 2 | 138 | 9.4% | 31.2% | 21.0% | 15.2% | 8.0% | 6.5% | 2.9% | 5.8% |
| Reopening 2 | 128 | 10.9% | 30.5% | 17.2% | 17.2% | 9.4% | 7.0% | 4.7% | 3.1% |
|  | Valid *N* | 0 times per week | 1 time per week | 2 times per week | 3 times per week | 4 times per week | 5 times per week | 6 times per week | 7 times per week |
| Savory snacks intake |  |  |  |  |  |  |  |  |  |
| Pre-pandemic | 674 | 7.4% | 61.9% | 20.9% | 6.8% | 2.2% | 0.3% | 0.0% | 0.4% |
| Lockdown 1 | 299 | 16.1% | 52.5% | 19.4% | 7.4% | 1.3% | 2.7% | 0.3% | 0.3% |
| Reopening 1 | 299 | 12.0% | 59.9% | 17.1% | 7.0% | 1.7% | 1.3% | 0.7% | 0.3% |
| Lockdown 2 | 138 | 29.7% | 54.3% | 12.3% | 1.4% | 0.7% | 0.7% | 0.7% | 0.0% |
| Reopening 2 | 128 | 28.9% | 56.2% | 9.4% | 3.1% | 1.6% | 0.0% | 0.0% | 0.8% |
|  | Valid *N* | 0 times per week | 1 time per week | 2 times per week | 3 times per week | 4 times per week | 5 times per week | 6 times per week | 7 times per week |
| Fruit and vegetables intake |  |  |  |  |  |  |  |  |  |
| Pre-pandemic | 674 | 0.1% | 0.1% | 1.9% | 4.0% | 9.1% | 16.3% | 22.3% | 46.1% |
| Lockdown 1 | 299 | 0.3% | 0.7% | 2.3% | 3.4% | 7.0% | 18.1% | 19.1% | 49.0% |
| Reopening 1 | 299 | 0.3% | 0.7% | 1.3% | 4.7% | 6.0% | 20.5% | 18.5% | 48.0% |
| Lockdown 2 | 138 | 0.0% | 0.0% | 2.2% | 4.3% | 7.2% | 13.8% | 24.6% | 47.8% |
| Reopening 2 | 128 | 0.0% | 0.0% | 0.8% | 1.6% | 9.4% | 23.4% | 22.7% | 42.2% |
|  | Valid *N* | Insufficiently active | Sufficiently active |  |  |  |  |  |  |
| Physical activity |  |  |  |  |  |  |  |  |  |
| Pre-pandemic | 557 | 29.1% | 70.9% |  |  |  |  |  |  |
| Lockdown 1 | 278 | 74.1% | 25.9% |  |  |  |  |  |  |
| Reopening 1 | 279 | 35.8% | 64.2% |  |  |  |  |  |  |
| Lockdown 2 | 138 | 65.9% | 34.1% |  |  |  |  |  |  |
| Reopening 2 | 128 | 40.5% | 59.4% |  |  |  |  |  |  |

*Note*. SSB = sugar-sweetened beverages.

**Supplemental Table E.**

*Correlations Between Adolescents’ Health Behaviors and Demographics.*

| Variable | Age^a^ | Educational type^b^ | Sex^c^ |
| --- | --- | --- | --- |
| Cigarette use |  |  |  |
| Pre-pandemic | .16*** | -.22*** | -.05 |
| Lockdown 1 | .20** | -.05 | .00 |
| Reopening 1 | .15* | -.06 | .04 |
| Lockdown 2 | .05 | .01 | .02 |
| Reopening 2 | .06 | -.08 | .01 |
| E-cigarette use |  |  |  |
| Pre-pandemic | .13** | -.16*** | -.08* |
| Lockdown 1 | .18** | -.14* | .04 |
| Reopening 1 | .19** | -.08 | .02 |
| Lockdown 2 | .11 | -.17* | .05 |
| Reopening 2 | -.04 | -.07 | -.08 |
| Alcohol use |  |  |  |
| Pre-pandemic | .26*** | -.13** | -.06 |
| Lockdown 1 | .20** | -.17** | -.04 |
| Reopening 1 | .24*** | -.12 | -.02 |
| Lockdown 2 | .20* | .01 | -.06 |
| Reopening 2 | .22* | .04 | .05 |
| SSB intake |  |  |  |
| Pre-pandemic | .08* | -.18*** | -.13** |
| Lockdown 1 | -.07 | -.11* | -.10 |
| Reopening 1 | -.04 | -.17** | -.10 |
| Lockdown 2 | .02 | .10 | -.10 |
| Reopening 2 | .06 | -.02 | .00 |
| Sweet snack intake |  |  |  |
| Pre-pandemic | -.05 | -.04 | -.02 |
| Lockdown 1 | -.04 | .00 | .07 |
| Reopening 1 | .02 | -.10 | .01 |
| Lockdown 2 | .05 | .11 | .07 |
| Reopening 2 | .10 | -.01 | -.06 |
| Savory snack intake |  |  |  |
| Pre-pandemic | .09* | -.12** | -.19*** |
| Lockdown 1 | .07 | -.11 | -.12* |
| Reopening 1 | .08 | -.02 | -.19** |
| Lockdown 2 | .10 | .00 | -.15 |
| Reopening 2 | .04 | .06 | -.24** |
| Fruit and vegetables intake |  |  |  |
| Pre-pandemic | .02 | .25*** | .07 |
| Lockdown 1 | -.01 | .09 | .03 |
| Reopening 1 | .00 | .12* | .10 |
| Lockdown 2 | .07 | .11 | .01 |
| Reopening 2 | .00 | -.01 | -.05 |
| Physical activity |  |  |  |
| Pre-pandemic | .09* | .07 | -.04 |
| Lockdown 1 | .12* | -.01 | -.10 |
| Reopening 1 | .09 | .04 | -.05 |
| Lockdown 2 | .12 | -.12 | -.04 |
| Reopening 2 | .00 | .09 | .03 |

*Note.* SSB = sugar-sweetened beverages. ^a^Age at the date of participation at pre-pandemic. ^b^0 = Low educational type; 1 = High educational type. ^c^0 = male; 1 = female. **p* < .05. ***p* < .01. ****p* < .001.

**Supplemental Table F.**

*Odds Ratios of Demographic Covariates Predicting Class Membership of Adolescents Per Timepoint (N = 710).*

| Wave | Covariate | *b* | *SE* | *p* | *OR* | Confidence interval | |
| --- | --- | --- | --- | --- | --- | --- | --- |
|  |  |  |  |  |  | 2.5 % | 97.5 % |
| Pre-pandemic |  |  |  |  |  |  |  |
| Class 1^a^ | Age | 1.28 | 0.27 | < .001 | 3.60 | 2.13 | 6.07 |
|  | Lower educational type | 1.79 | 0.37 | < .001 | 6.00 | 2.91 | 12.35 |
|  | Male sex | 0.67 | 0.36 | .063 | 1.96 | 0.97 | 3.96 |
|  |  |  |  |  |  |  |  |
| Class 3^b^ | Age | 0.36 | 0.21 | .089 | 1.43 | 0.95 | 2.17 |
|  | Lower educational type | 1.20 | 0.25 | < .001 | 3.33 | 2.04 | 5.43 |
|  | Male sex | 0.62 | 0.24 | .010 | 1.90 | 1.16 | 2.99 |
| Lockdown 1 |  |  |  |  |  |  |  |
| Class 1^a^ | Age | -0.55 | 0.43 | .200 | 0.58 | 0.25 | 1.34 |
|  | Lower educational type | 0.34 | 0.58 | .563 | 1.40 | 0.45 | 4.35 |
|  | Male sex | -0.17 | 0.55 | .757 | 0.84 | 0.29 | 2.47 |
| Reopening 1 |  |  |  |  |  |  |  |
| Class 1^a^ | Age | 0.95 | 0.47 | .045 | 2.58 | 1.02 | 6.54 |
|  | Lower educational type | -0.36 | 1.18 | .758 | 0.70 | 0.07 | 7.07 |
|  | Male sex | 1.22 | 1.23 | .320 | 3.40 | 0.31 | 37.82 |
| Lockdown 2 |  |  |  |  |  |  |  |
| Class 1^a^ | Age | 0.25 | 0.42 | .546 | 1.28 | 0.57 | 2.90 |
|  | Lower educational type | -0.12 | 0.63 | .846 | 0.89 | 0.26 | 3.02 |
|  | Male sex | -0.07 | 0.58 | .904 | 0.93 | 0.30 | 2.92 |
| Reopening 2 |  |  |  |  |  |  |  |
| Class 1^a^ | Age | 0.27 | 0.35 | .437 | 1.31 | 0.66 | 2.61 |
|  | Lower educational type | 0.37 | 0.59 | .529 | 1.45 | 0.46 | 4.59 |
|  | Male sex | -0.83 | 0.71 | .241 | 0.44 | 0.11 | 1.74 |

*Note.* The classes with health-protective substance use and food intake (Class 2) are not present because they were used as reference class in the logistic regression. ^a^Class with health-risk substance use and food intake. ^b^Medium health-risk class (at pre-pandemic only). For the binary variables educational level and sex, higher education and females was used as reference class, respectively. OR = odds ratio.

**Supplemental Table G.**

*Multinomial Logistic Regressions Predicting Class Membership by Parental Health Behaviors at Pre-Pandemic (Univariate Analyses; N = 334 to 378).*

|  |  | *b* | *SE* | *p* | | Nagelkerke *R^2d^* | *OR* | Confidence interval | | |
| --- | --- | --- | --- | --- | --- | --- | --- | --- | --- | --- |
|  |  |  |  |  |  |  |  | 2.5 % | | 97.5 % |
| Class 1^a^ | Cigarette use^c^ |  |  | |  |  |  |  |  | |
|  | Experimental | -0.31 | 0.50 | | .529 |  | 0.73 | 0.27 | 1.95 | |
|  | Infrequent current^d^ | - | - | | - |  | - | - | - | |
|  | Frequent current | -0.15 | 0.79 | | .849 |  | 0.86 | 0.18 | 4.10 | |
|  | Alcohol use^e^ |  |  | |  |  |  |  |  | |
|  | Experimental | -0.81 | 0.79 | | .304 |  | 0.44 | 0.09 | 2.09 | |
|  | Infrequent current | -0.73 | 0.59 | | .221 |  | 0.48 | 0.15 | 1.55 | |
|  | Frequent current | -0.60 | 0.64 | | .351 |  | 0.55 | 0.16 | 1.94 | |
|  | SSBs intake | 0.08 | 0.09 | | .372 |  | 1.08 | 0.91 | 1.29 | |
|  | Sweet snack intake | 0.14 | 0.12 | | .226 |  | 1.15 | 0.92 | 1.45 | |
|  | Savory snack intake | 0.01 | 0.41 | | .982 |  | 1.01 | 0.45 | 2.25 | |
|  | Fruit & veg intake | -0.23 | 0.19 | | .215 |  | 0.79 | 0.55 | 1.14 | |
|  | Physical activity^f^ | -0.25 | 0.60 | | .678 |  | 0.78 | 0.24 | 2.54 | |
| Class 3^b^ | Cigarette use^c^ |  |  | |  | 0.26 |  |  |  | |
|  | Experimental | -0.36 | 0.26 | | .157 |  | 0.70 | 0.42 | 1.15 | |
|  | Infrequent current^d^ | - | - | | - |  | - | - | - | |
|  | Frequent current | -0.09 | 0.40 | | .823 |  | 0.91 | 0.41 | 2.01 | |
|  | Alcohol use^e^ |  |  | |  | 0.25 |  |  |  | |
|  | Experimental | -0.48 | 0.46 | | .292 |  | 0.62 | 0.25 | 1.52 | |
|  | Infrequent current | -0.06 | 0.36 | | .864 |  | 0.94 | 0.46 | 1.91 | |
|  | Frequent current | 0.04 | 0.38 | | .907 |  | 1.05 | 0.49 | 2.22 | |
|  | SSBs intake | 0.09 | 0.05 | | .071 | 0.23 | 1.09 | 0.99 | 1.20 | |
|  | Sweet snack intake | 0.02 | 0.07 | | .799 | 0.22 | 1.02 | 0.89 | 1.16 | |
|  | Savory snack intake | 0.65 | 0.21 | | .003 | 0.26 | 1.92 | 1.26 | 2.93 | |
|  | Fruit & veg intake | -0.36 | 0.10 | | <.001 | 0.26 | 0.70 | 0.58 | 0.85 | |
|  | Physical activity^f^ | -0.12 | 0.29 | | .682 | 0.46 | 0.89 | 0.50 | 1.57 | |

*Note.* The class with health-protective substance use and food intake (Class 2) is not present because it is used as reference class in the logistic regression. ^a^Class with health-risk substance use and food intake. ^b^Medium health-risk class (at pre-pandemic only). ^c^Reference level for cigarette use was non-smoking. ^d^Estimates for this level were not reliable because very few parents were infrequent current users. ^e^Reference level for alcohol use was non-drinking. All the food intake variables were continuous. ^f^Effect of sufficiently active versus insufficiently active. E-cigarette use is not present since almost no parents indicated use of e-cigarettes. OR = odds ratio.

**Supplemental Table H.**

*Logistic Regressions Predicting Class Membership by Parental Health Behaviors at Lockdown 1 (Univariate Analyses; N = 156 to 162).*

|  | *b* | *SE* | *p* | Nagelkerke *R^2^* | *OR* | Confidence Interval | |
| --- | --- | --- | --- | --- | --- | --- | --- |
|  |  |  |  |  |  | 2.5% | 97.5% |
| Cigarette use^a^ |  |  |  | 0.01 |  |  |  |
| Experimental | 0.49 | 1.24 | .691 |  | 1.64 | 0.07 | 17.61 |
| Infrequent current | 1.19 | 1.43 | .406 |  | 3.27 | 0.13 | 84.29 |
| Frequent current | 0.49 | 0.64 | .444 |  | 1.64 | 0.42 | 5.55 |
| Alcohol use^b^ |  |  |  | 0.03 |  |  |  |
| Experimental | 0.38 | 0.63 | .540 |  | 1.47 | 0.43 | 5.16 |
| Infrequent current | -0.62 | 0.57 | .269 |  | 0.54 | 0.18 | 1.67 |
| Frequent current | -0.05 | 0.54 | .921 |  | 0.95 | 0.33 | 2.87 |
| SSBs intake (continuous) | 0.10 | 0.08 | .175 | 0.02 | 1.11 | 0.95 | 1.29 |
| Sweet snack intake (continuous) | 0.14 | 0.09 | .149 | 0.02 | 1.15 | 0.95 | 1.38 |
| Savory snack intake (continuous) | 0.46 | 0.30 | .126 | 0.02 | 1.58 | 0.88 | 2.89 |
| Fruit & veg intake (continuous) | -0.24 | 0.15 | .096 | 0.02 | 0.78 | 0.59 | 1.05 |
| Physical activity^c^ | -0.48 | 0.40 | .220 | 0.01 | 0.62 | 0.29 | 1.36 |

*Note.* The class with health-protective substance use and food intake is used as reference class in the logistic regression. ^a^Reference level for cigarette use was non-smoking. ^b^Reference level for alcohol use was non-drinking. All the food intake variables were continuous. ^c^Effect of sufficiently active versus insufficiently active. E-cigarette use is not present since almost no parents indicated use of e-cigarettes. OR = odds ratio.

**Supplemental Table I.**

*Logistic Regressions Predicting Class Membership by Parental Health Behaviors at Reopening 1 (Univariate Analyses; N = 156 to 162).*

|  | *b* | *SE* | *p* | Nagelkerke *R^2^* | *OR* | Confidence Interval | |
| --- | --- | --- | --- | --- | --- | --- | --- |
|  |  |  |  |  |  | 2.5% | 97.5% |
| Cigarette use^a^ |  |  |  | 0.01 |  |  |  |
| Experimental | 0.04 | 1.17 | .974 |  | 1.04 | 0.05 | 8.42 |
| Infrequent current | 1.14 | 1.43 | .426 |  | 3.12 | 0.12 | 80.27 |
| Frequent current | 0.04 | 0.70 | .956 |  | 1.04 | 0.22 | 3.71 |
| Alcohol use^b^ |  |  |  | 0.00 |  |  |  |
| Experimental | 0.47 | 0.70 | .499 |  | 1.60 | 0.41 | 6.53 |
| Infrequent current | 0.31 | 0.58 | .594 |  | 1.36 | 0.46 | 4.66 |
| Frequent current | 0.29 | 0.59 | .628 |  | 1.33 | 0.43 | 4.63 |
| SSBs intake (continuous) | 0.19 | 0.08 | .011 | 0.06 | 1.21 | 1.04 | 1.40 |
| Sweet snack intake (continuous) | 0.07 | 0.10 | .485 | 0.00 | 1.07 | 0.88 | 1.29 |
| Savory snack intake (continuous) | 0.13 | 0.29 | .664 | 0.00 | 1.14 | 0.63 | 2.02 |
| Fruit & veg intake (continuous) | -0.26 | 0.15 | .078 | 0.03 | 0.77 | 0.57 | 1.03 |
| Physical activity^c^ | 0.23 | 0.43 | .600 | 0.00 | 1.25 | 0.55 | 3.07 |

*Note.* The class with health-protective substance use and food intake is used as reference class in the logistic regression. ^a^Reference level for cigarette use was non-smoking. ^b^Reference level for alcohol use was non-drinking. All the food intake variables were continuous. ^c^Effect of sufficiently active versus insufficiently active. E-cigarette use is not present since almost no parents indicated use of e-cigarettes. OR = odds ratio.

**Supplemental Table J.**

*Logistic Regressions Predicting Class Membership by Parental Health Behaviors at Lockdown 2 (Univariate Analyses; N = 96 to 98).*

|  | *b* | *SE* | *p* | Nagelkerke *R^2^* | *OR* | Confidence Interval | |
| --- | --- | --- | --- | --- | --- | --- | --- |
|  |  |  |  |  |  | 2.5% | 97.5% |
| Cigarette use^a^ |  |  |  | 0.00 |  |  |  |
| Experimental | -0.12 | 0.63 | .854 |  | 0.89 | 0.23 | 2.88 |
| Infrequent current^b^ | - | - | - |  | - | - | - |
| Frequent current | -0.32 | 1.15 | .778 |  | 0.72 | 0.04 | 5.28 |
| Alcohol use^c^ |  |  |  | 0.11 |  |  |  |
| Experimental | -1.19 | 1.23 | .333 |  | 0.30 | 0.01 | 2.82 |
| Infrequent current | -0.21 | 0.76 | .782 |  | 0.81 | 0.20 | 4.16 |
| Frequent current | 0.96 | 0.77 | .212 |  | 2.62 | 0.62 | 13.85 |
| SSBs intake (continuous) | -0.14 | 0.11 | .201 | 0.03 | 0.87 | 0.68 | 1.06 |
| Sweet snack intake (continuous) | -0.21 | 0.15 | .175 | 0.03 | 0.81 | 0.58 | 1.08 |
| Savory snack intake (continuous) | -0.33 | 0.43 | .448 | 0.00 | 0.72 | 0.30 | 1.65 |
| Fruit & veg intake (continuous) | -0.33 | 0.19 | .077 | 0.05 | 0.72 | 0.49 | 1.03 |
| Physical activity^d^ | 0.50 | 0.47 | .288 | 0.02 | 1.65 | 0.65 | 4.26 |

*Note.* The class with health-protective substance use and food intake is used as reference class in the logistic regression. All the food intake variables were continuous. ^a^Reference level for cigarette use was non-smoking. ^b^Estimates for this level were not reliable because very few parents were infrequent current users. ^c^Reference level for alcohol use was non-drinking. ^d^Effect of sufficiently active versus insufficiently active. E-cigarette use is not present since almost no parents indicated use of e-cigarettes. OR = odds ratio.

**Supplemental Table K.**

*Logistic Regressions Predicting Class Membership by Parental Health Behaviors at Reopening 2 (Univariate Analyses; N = 88).*

|  | *b* | *SE* | *p* | Nagelkerke *R^2^* | *OR* | Confidence Interval | |
| --- | --- | --- | --- | --- | --- | --- | --- |
|  |  |  |  |  |  | 2.5% | 97.5% |
| Cigarette use^a^ |  |  |  |  |  |  |  |
| Experimental | - | - | - |  | - | - | - |
| Infrequent current | - | - | - |  | - | - | - |
| Frequent current | - | - | - |  | - | - | - |
| Alcohol use^b^ |  |  |  | 0.08 |  |  |  |
| Experimental | 0.03 | 0.84 | .973 |  | 1.03 | 0.19 | 5.41 |
| Infrequent current | 0.20 | 0.65 | .753 |  | 1.23 | 0.35 | 4.67 |
| Frequent current | 1.22 | 0.69 | .078 |  | 3.40 | 0.90 | 14.16 |
| SSBs intake (continuous) | 0.01 | 0.10 | .912 | 0.00 | 1.01 | 0.83 | 1.24 |
| Sweet snack intake (continuous) | -0.18 | 0.12 | .120 | 0.04 | 0.83 | 0.65 | 1.04 |
| Savory snack intake (continuous) | -0.26 | 0.30 | .382 | 0.01 | 0.77 | 0.39 | 1.29 |
| Fruit & veg intake (continuous) | 0.13 | 0.20 | .527 | 0.00 | 1.13 | 0.77 | 1.70 |
| Physical activity^c^ | 0.55 | 0.45 | .214 | 0.02 | 1.74 | 0.73 | 4.24 |

*Note.* The class with health-protective substance use and food intake is used as reference class in the logistic regression. All the food intake variables were continuous. ^a^No estimates are provided for cigarette use due to sparseness, which resulted in an unreliable model. ^b^Reference level for alcohol use was non-drinking. ^c^Effect of sufficiently active versus insufficiently active. E-cigarette use is not present since almost no parents indicated use of e-cigarettes. OR = odds ratio.
